# Prenatal exposure to acrylamide and metabolic health at 20 years of age A biomarker-based Danish cohort study

**DOI:** 10.1016/j.envint.2025.110000

**Published:** 2026-01

**Authors:** Stéphane Tuffier, Efstathios Vryonidis, Thorhallur Ingi Halldorsson, Anne Ahrendt Bjerregaard, Damian Chandia-Poblete, Dorte Rytter, Bodil Hammer Bech, Tine Brink Henriksen, Thorkild I.A. Sørensen, Sjurdur Frodi Olsen, Margareta Törnqvist, Marie Pedersen

**Affiliations:** a Department of Public Health, University of Copenhagen, Copenhagen, Denmark; b Department of Environmental Science, Stockholm University, Stockholm, Sweden; c Department for Congenital Disorders, Statens Serum Institut, Copenhagen, Denmark; d Faculty of Food Science and Nutrition, University of Iceland, Reykjavik, Iceland; e Centre for Clinical Research and Prevention, Copenhagen University Hospital - Bispebjerg and Frederiksberg Hospital, Copenhagen, Denmark; f Department of Public Health, Aarhus University, Denmark; g Department of Paediatrics, Aarhus University Hospital, Skejby, Denmark; h Novo Nordisk Foundation Center for Basic Metabolic Research, Genomic Physiology and Translation, NNF CBMR, Copenhagen, Denmark; i Department of Nutrition, Harvard School of Public Health, Boston, USA

**Keywords:** Obesity, Metabolic Diseases, Acrylamide, Biomarkers, Developmental Origins of Health and Disease, Diet, Tobacco smoking

## Abstract

Adult obesity and metabolic health may be influenced by prenatal exposure to acrylamide (AA). AA forms in carbohydrate-containing foods and beverages during high-temperature processing. AA disrupts metabolic homeostasis and induces adiposity in mice offspring following gestational exposure. In humans, AA has been associated with intrauterine growth restriction and childhood overweight. It is unknown if prenatal exposure to AA influences metabolic health in young adults. We examined the associations between prenatal exposure to AA and metabolic health in singletons born in 1988–1989, Denmark.

Hemoglobin adducts from AA (HbAA) and glycidamide were measured in maternal blood collected at 30 weeks’ gestation together with information on maternal dietary and smoking habits (n = 638). At 20 years of age, offspring waist circumference, weight, height, blood pressure, blood glucose, insulin, leptin, adiponectin and lipid levels were measured at a clinical follow-up.

Median HbAA concentration was 85 (interquartile range 63–136) pmol/g Hb. For most outcomes examined, there was no evidence of an association with prenatal exposure to AA. However, higher HbAA levels were associated with a minor increase in the offspring waist circumference of 0.06 cm (95 % confidence interval (CI): -0.07, 0.20) and low-density lipoprotein (LDL) cholesterol concentrations of 0.01 mmol/L (95 % CI: <0.01, 0.03) per 10 pmol/g Hb increments in HbAA. Furthermore, we observed that HbAA levels were associated with increased risk of metabolic syndrome in offspring of non-smokers.

We found no overall compelling evidence that prenatal exposure to AA was associated with metabolic health of young adults, but the observations that higher prenatal exposure to AA were associated with higher waist circumference and LDL cholesterol levels in young adults warrants replication.

## Highlights

• Hemoglobin adducts of AA measured in pregnant women reflect prenatal exposure to AA from multiple sources.
• Prenatal exposure to AA were associated with higher waist circumference and LDL cholesterol levels at 20 years of age.
• Absence of associations between prenatal AA and other metabolic health outcomes.
• More studies are needed to understand the potential role of prenatal exposure to AA in adult health.

## 1 Introduction

Acrylamide (AA) is formed in many commonly consumed carbohydrate-containing food and beverages e.g. potato chips, fried potatoes, crisp bread, biscuits, breakfast cereals, dried fruits, and coffee as a result of frying, baking or roasting at high temperatures (European Food Safety Agency [EFSA], Panel on Contaminants in the Food Chain (CONTAM), 2015, Tareke et al., 2002). It is primarily formed through the Maillard reaction between reducing sugars and the amino acid asparagine in plant-based foods (Mottram et al. 2002). Following the discovery of AA in food, evidence from the extensive food monitoring programs implemented in multiple countries worldwide demonstrate that AA is ubiquitous in multiple foods and beverages (Abt et al. 2019). Hence, diet is the major source of exposure to AA in the general population with tobacco smoking being the second most common source of exposure (Smith et al. 2000). The concentrations of AA in foods and beverages vary greatly even within identical food items as its formation depends on multiple factors, such as the composition of the food, cooking methods, temperature, duration of cooking, storage conditions, moisture or the pH (European Food Safety Agency [EFSA], Panel on Contaminants in the Food Chain (CONTAM), 2015, Pedersen et al., 2022, Riboldi et al., 2014, Timmermann et al., 2021). This makes it difficult to estimate dietary AA intake using food-frequency questionnaires or dietary records. Nevertheless, dietary exposure to AA is estimated to be widespread with a median dietary AA intake ranging from 0.02 to 1.53 μg/kg body weight/day according to published calculations based on food monitoring and individual food intake data from multiple countries (Timmermann et al. 2021).

AA has recently been proposed to act as an obesogen through multiple biological mechanisms (Amato et al., 2021, Buyukdere and Akyol, 2023). For instance, AA disrupts the metabolic homeostasis and induces adiposity in mice offspring following gestational exposure (Lee and Pyo 2019). Hemoglobin adducts from AA (HbAA) measured in human maternal blood or cord blood and calculations of maternal dietary intake of AA from food-frequency questionnaires obtained in mid-pregnancy were used to estimate prenatal exposure to AA and consistently shown to be associated with lower birth weight (Chandia-Poblete et al., 2025, Duarte-Salles et al., 2013, Hogervorst et al., 2021; 2022; Kadawathagedara et al., 2016, Nagata et al., 2019, Pedersen et al., 2012, Zhan et al., 2020), a risk factor for obesity and metabolic disorders (Cauzzo et al., 2023, Oken and Gillman, 2003). Maternal dietary intake of AA in pregnancy estimated with food-frequency questionnaires has also been associated with higher risk of overweight/obesity in the offspring during early childhood (Kadawathagedara et al. 2018), but these studies did not address whether prenatal exposure to AA increases the risk of overweight/obesity as well as other markers of poor metabolic health in early adulthood.

In this study, we examined the associations between prenatal exposure to AA and metabolic offspring health measured at 20-years-of age in a birth cohort study from Denmark. HbAA and Hb adducts of its metabolite glycidamide (GA) were used as biomarkers of exposure to AA.

## 2 Methods

### 2.1 Study population

This study relied on data collected as part of the Danish Fetal Origins 1988–89 (DaFO88) birth cohort (Olsen et al. 1995b). Briefly, between April the 15th 1988 and January the 15th 1989, women with singleton pregnancy were recruited from a large antenatal clinic at Aarhus Hospital, Denmark. Eligible women were scheduled for routine visit in gestational week 30. One week before the visit, a self-administered dietary questionnaire was mailed to the women. Out of 1,212 eligible women, 991 (82 %) were enrolled in the study. Following informed consent, 965 women participated in a face-to-face interview and gave information about diet, smoking in pregnancy, anthropometry, medical and pregnancy history, and socioeconomic status in terms of cohabitation, education and income. Information about the pregnancy, and the newborn was collected from the medical records after birth and from the Danish Medical Birth Registry (Knudsen and Olsen 1998). Women were asked to report dietary habits in a 3-month time window back in time (Bjerregaard et al., 2019, Olsen et al., 1995a). Following principles described earlier (Bjerregaard et al. 2019), we estimated maternal diet quality by calculating the Healthy Eating Index (HEI), as a score ranging from 0 to 80, based on 8 diet components, with 80 as complete adherence to the Danish Food-Based Dietary Guidelines (Supplemental Materials).

A peripheral maternal blood sample was obtained after the interview at gestational week 30. For a subset of women, an additional blood sample was also drawn at 37 gestational weeks. The erythrocytes were isolated and stored at –20 °C.

### 2.2 Assessment of prenatal exposure to acrylamide

Hb adduct levels (pmol/g Hb) were determined by the “FIRE procedure” (fluorescein isothiocyanate R Edman). For the present study the method was further developed formodified for higher throughput analysis (Vryonidis et al. 2024). The principle of the method is that adducted N-terminal valines are detached using the Edman reagent fluoresceine-5-isothiocyanate forming fluorescein hydantoin (FTH) derivatives which are analyzed by liquid chromatography − mass spectrometry (LC-MS). “FIRE procedure” has previously been applied for analysis of prenatal exposure to AA by us (Chandia-Poblete et al. 2025; Pedersen et al., 2012, Von Stedingk et al., 2010, Von Stedingk et al., 2011).

In this study samples of 120 µL of whole blood were added to 96 deep well plates. After dilution (240 µL water) and mixing (2 min at 1500 rpm), 20 µL of each sample was taken out for measurement of the concentration of Hb with the sodium lauryl sulfate method as earlier described (Vryonidis et al. 2024). Deuterium-substituted internal standards were added, and after mixing the Edman reagent was added, next samples were derivatized overnight at 25 °C and 500 rpm. After protein precipitation with acetonitrile, the detached FTH analytes were isolated and extracted by solid phase extraction and analyzed by LC-MS. Triplicate of three quality control (QC) blood samples with different adduct levels were used in each batch. Quantification was done using calibration curves, prepared from adduct-modified peptides added to human blood samples as described earlier.

We analyzed blood for the FTH analytes of HbAA and HbGA adducts in 896 maternal samples collected at 30 gestational weeks and 73 samples at 37 weeks. For 3 women, only samples from 37 gestational weeks were available and used. In total, 899 women had at least one measure of HbAA.

The HbAA adduct levels were quantified in 100 % and the HbGA adduct levels in 39 % of the total number (n = 370). The Supplemental Material Fig. S1 shows the flow chart of the study populations.

Overall, the quantification frequency of the HbGA adduct analyte was lower than for the HbAA adduct analyte. This was due to difficulties with the current state of the analytical method with respect to the FTH analyte of HbGA. This analyte had lower overall signal-to-noise ratio and is appearing as broad double peak, which is due to GA being an asymmetric epoxide that forms two reaction products. Sample repeatability of the AA analyte was assessed using the quality control samples. The relative standard deviation of all QC samples was on average 12 %.

Furthermore, Hb adduct levels from ethylene oxide (HbEO) were measured in 826 blood samples to assess exposure to active and second-hand tobacco smoke during pregnancy using the FIRE procedure (cf. Von Stedingk et al. 2011).

### 2.3 Assessment of offspring metabolic health

In 2008–2009, a total of 915 mothers and their offspring were identified in the Danish registers and the offspring for whom a mother was identified were contacted (Halldorsson et al., 2012, Rytter et al., 2013). The offspring were asked to complete an online questionnaire concerning their current health, lifestyle including smoking and dietary habits, and their current height and weight. In addition, a tape measure was mailed to all potential participants with instructions on how to measure their waist circumference. Offspring were also invited to participate in a 75–100 min clinical examination, which included height, weight and waist circumference measurements taken by a nurse and collection of a fasting blood sample that was immediately centrifuged and stored at –80 °C.

### 2.4 Anthropometric outcomes

Offspring height, weight, and waist circumference were first extracted from the clinical examinations (n = 400). For offspring who did not attend the clinical examination, self-measurements were used (n = 238). Missing observations (n ≤ 3) in offspring’s weight, waist circumference, and height were imputed using robust regression with an M estimator as described in the statistical analysis section (van Buuren, 2018).

A total of 638 offspring of mothers with available HbAA and 241 offspring of mothers with HbGA had complete information on anthropometric measures (Supplemental Material, Fig S1).

Body mass index (BMI) was calculated for each offspring and dichotomized in underweight and normal BMI (<25 kg/m^2^) or overweight or obese (≥25 kg/m^2^). Large waist circumference was considered as having a waist circumference > 102 cm for male and > 88 cm for female offspring (Beilby 2004).

### 2.5 Metabolic biomarkers

At the clinical examination, systolic blood pressure (SBP), diastolic blood pressure (DBP) and blood glucose levels were measured. After a rest period of 5 min, lying on an examination bench, participant’s SBP and DBP were measured 3 times with an automatic device ((OMRON M6 Comfort (HEM-7000-E))(Rytter et al. 2013). We averaged the three measures. Blood glucose was measured with an Accu-Chek device. Metabolic biomarkers related to adiposity were measured in the fasting blood samples and included insulin, leptin, adiponectin and lipids profile (total cholesterol, low-density lipoproteins (LDL) cholesterol, and high-density lipoproteins (HDL)). Adiponectin was measured by a time-resolved immunofluorometric assay based on two antibodies and recombinant human adiponectin (RD Systems, Abingdon, United Kingdom) as previously described (Frystyk et al. 2005). Leptin was determined by a time-resolved immunofluorometric assay based on commercial reagents (R&D Systems) using recombinant human leptin as the standard; otherwise, measurements were carried out essentially as for adiponectin (Frystyk et al. 2005). Plasma insulin concentrations were determined by the commercial Insulin ELISA kit (Dako, Copenhagen, Denmark).

A total of 400 offspring of mothers with available HbAA and 148 offspring of mothers with HbGA had complete information on metabolic biomarkers.

Metabolic syndrome was defined by one or more of the following ATP III criteria (Beilby 2004): a waist circumference > 102 cm for males and > 88 cm for females; HDL cholesterol < 1.04 mmol/L for males and < 1.30 mmol/L for females; triglycerides ≥ 1.7 mmol/L, systolic blood pressure ≥ 130 mmHg or diastolic blood pressure ≥ 85 mmHg, or fasting blood sugar ≥ 6.1 mmol/L.

Insulin resistance (HOMA-IR) and β-cell function (HOMA-B) were calculated according to homeostasis model assessment formulas: HOMA-IR = (insulin * blood sugar / 22.5) * 0.114, HOMA-B = ((insulin * 20) / (blood sugar − 3.5)) * 0.114 (Matthews et al. 1985).

The DaFO88 protocols were approved by the Regional Scientific Ethical Committee of the county of Aarhus, Denmark and the Scientific Ethical Committee of the Capital Region of Denmark (reference no. 20070157 and H-19015014). All participating pregnant women and offspring at the 20 years follow-up provided written consent prior to participation in the studies.

The biomarker study of exposure to AA was also approved by the Swedish Regional Ethical Authority (no. 2019–03181).

### 2.6 Statistical analysis

We report descriptive results for the whole study population and after stratification by maternal smoking status to isolate active smoking from other sources of AA such as diet. Associations between prenatal AA exposure and offspring metabolic health were analyzed using multiple linear regression for continuous outcomes and multiple logistic regression for binary outcomes. Insulin, leptin and adiponectin levels were natural log transformed to correct for non-normal distributions. We entered the Hb adduct levels as a continuous scale variable (effect estimated per 10 pmol/g Hb increments) or as categorized according to quartiles of the distribution. We fitted HbAA and HbGA adduct levels separately one-by-one in the models. We present results for HbGA in the Supplemental Materials as HbAA levels were our main exposure of interest. We assessed shape of the potential dose–response associations of HbAA and HbGA with the metabolic outcomes by using discrete variable of exposure, categorized in quartiles; and by evaluating the graphs of natural cubic splines with 2 to 6 degrees of freedom. Splines with the lowest Akaike information criterion (AIC) were retained.

We identified covariates from literature and biological understanding and used a directed acyclic graph (DAG) to assist the selection (Supplemental Material, Fig. S2). Maternal smoking and diet were thought to be important confounders, and three types of models were calculated to investigate their potential influence. We present models without adjustment for smoking and diet as these are sources of AA and it can be argued that adjustment may be overadjustment. Model 1 was adjusted for maternal age at pregnancy (continuous), maternal education (Other or no education, Short education, Intermediate education or Academic education) and maternal annual household income (<150,000, 150,000 to 200,000, 200,000 to 300,000, > 300,000 DKK/year); Model 2 was further adjusted for maternal smoking during pregnancy (any time, yes or no); Models 3 (final model) were further adjusted for maternal healthy eating index (HEI, continuous). Models 4 were further adjusted for maternal overweight or obesity as indicator of maternal metabolic status, which was considered as a potential mediator and therefore not included in the main models. Missing covariate observations were imputed using either classification regression tree model or robust regression using an M estimator (van Buuren, 2018).

We performed several sensitivity analyses by 1) stratifying analysis on maternal smoking status, maternal education level and offspring sex; 2) using only complete case data to evaluate sensitivity to imputation; 3) stratifying analysis of anthropometrics measurement on offspring with and without clinical examination; 4) repeating the analysis using sex specific z-scores of all continuous outcomes calculated among male or female offspring; 5) using the sum of HbAA and HbGA as the exposure.

We stratified association analyses by sex as growth, metabolism and metabolic health differ between sex (Dearden et al. 2018). Furthermore, we stratified association analyses by maternal education in order to evaluate if the associations between prenatal exposure to AA and metabolic health differ across offspring of different levels of SES, and we hypothesized that offspring of women with low education could be more vulnerable due to unmeasured co-exposure to factors that may correlate with prenatal exposure to AA and metabolic health than those of women of higher education.

Univariate tests were performed using double-sided Student’s t-Test or Pearson’s Chi-squared test. Correlations between Hb adduct levels were tested using Pearson correlation coefficients. Statistical significance was defined as a two-sided p < 0.05 for all the tests. Adjusted point estimates of the associations are provided with 95 % confidence intervals (CI). All analyses were performed using R with tidyverse (Wickham et al. 2019) and targets (Landau 2021) packages on the secure servers provided by Statistics Denmark. The study code is available online: https://osf.io/eh8jy/.

## 3 Results

### 3.1 Study population characteristics

Table 1 summarizes the characteristics of the full study population with anthropometric measures (n = 638) and of the subpopulation with metabolic biomarkers measures (n = 400) by maternal smoking status in pregnancy. The mean (± SD) maternal age at inclusion was 29.2 ± 4.1 years, with most mothers having intermediate or high education levels as well as high income. A total of 413 (64.7 %) of the mothers were classified as non-smokers during pregnancy and among them 388 (93.9 %) had a normal weight pre-pregnancy. Non-smoking mothers had higher education, but a slightly higher BMI than smokers. The mean HEI was 35 ± 7 points, with non-smoking mothers eating healthier than smokers (35 ± 7 vs 34 ± 7 points).

**Table 1:** Study population characteristics by maternal smoking status.

	All (n = 638)	Maternal Smoking		
		No (n = 413)	Yes (n = 225)	p-value
Maternal characteristics				
Maternal age (years), mean ± SD	29.2 ± 4.1	29.4 ± 4.1	28.8 ± 4	0.07
Length of education, n (%)				<0.001
Other or no education	60 (9.4)	37 (9)	23 (10.2)	
Short education	211 (33.1)	110 (26.6)	101 (44.9)	
Intermediate education	260 (40.8)	193 (46.7)	67 (29.8)	
Academic education	107 (16.8)	73 (17.7)	34 (15.1)	
Household income (DKK/year), n (%)				0.24
<150,000	104 (16.3)	67 (16.2)	37 (16.4)	
150,000 to 200,000	115 (18)	72 (17.4)	43 (19.1)	
200,000 to 300,000	212 (33.2)	129 (31.2)	83 (36.9)	
> 300,000	207 (32.4)	145 (35.1)	62 (27.6)	
Healthy eating index, mean ± SD	34.6 ± 6.8	35.1 ± 6.6	33.7 ± 7.1	0.011
Pre-pregnancy BMI (kg/m^2^), mean ± SD	21.3 ± 2.9	21.4 ± 3	21.2 ± 2.6	0.43
Overweight or Obese, n (%)	50 (7.8)	35 (8.5)	15 (6.7)	0.005
Smoking pregnancy, n (%)	225 (35.3)			
HbAA (pmol/g Hb), mean ± SD	108.7 ± 68.8	83.3 ± 42.3	155.4 ± 82.5	< 2e-16
HbGA (pmol/g Hb), mean ± SD	166.3 ± 111.3	101.6 ± 71.6	213.1 ± 111.5	< 2e-16
HbEO (pmol/g Hb), mean ± SD	136.1 ± 137.7	82.6 ± 76.5	234.3 ± 168.0	< 2e-16

Offspring’s characteristics				
Sex, n (%)				0.36
Female	309 (48.4)	206 (49.9)	103 (45.8)	
Male	329 (51.6)	207 (50.1)	122 (54.2)	

	**Anthropometric outcomes**			
Weight (kg), mean ± SD	69.8 ± 12.4	69.6 ± 12	70 ± 13.1	0.75
Height (cm), mean ± SD	175.8 ± 9.5	176.4 ± 9.4	174.8 ± 9.8	0.048
Waist circumference (cm), mean ± SD	82.1 ± 9.5	81.4 ± 8.7	83.3 ± 10.8	0.022
Large waist circumference, n (%)	62 (9.7)	29 (7)	33 (14.7)	0.61
BMI (kg/m^2^), mean ± SD	22.5 ± 3.1	22.3 ± 2.9	22.8 ± 3.3	0.052
Overweight or Obese, n (%)	116 (18.2)	66 (16)	50 (22.2)	0.14

	**Metabolic biomarkers**	**N = 400**	**N = 255**	**N = 145**
Systolic blood pressure (mmHg), mean ± SD	110.7 ± 10.9	110.3 ± 10.7	111.4 ± 11.2	0.33
Diastolic blood pressure (mmHg), mean ± SD	65.9 ± 6.6	65.4 ± 6.6	66.7 ± 6.4	0.06
Triglycerides (mmol/L), mean ± SD	1 ± 0.4	1 ± 0.4	1 ± 0.5	0.53
Total cholesterol (mmol/L), mean ± SD	4.4 ± 0.9	4.4 ± 0.9	4.4 ± 0.8	0.65
LDL cholesterol (mmol/L), mean ± SD	2.5 ± 0.7	2.4 ± 0.7	2.5 ± 0.7	0.49
HDL cholesterol (mmol/L), mean ± SD	1.5 ± 0.3	1.5 ± 0.3	1.5 ± 0.3	0.56
Blood sugar (mmol/L), mean ± SD	4.9 ± 0.5	4.9 ± 0.5	4.9 ± 0.4	0.68
Insulin (pmol/L), mean ± SD	43.5 ± 21.6	43 ± 23.4	44.5 ± 18.1	0.49
Leptin (μg/L), mean ± SD	11.8 ± 12.4	11.3 ± 11.9	12.7 ± 13.3	0.31
Adiponectin (mg/L), mean ± SD	8.9 ± 3.7	8.7 ± 3.5	9.4 ± 3.9	0.077
HOMA IR index, mean ± SD	1.4 ± 0.8	1.4 ± 0.8	1.4 ± 0.6	0.59
HOMA β Index, mean ± SD	95.4 ± 62.7	92.4 ± 58.9	100.8 ± 68.8	0.22
Metabolic syndrome, n (%)	140 (35)	82 (32.2)	58 (40)	0.043

Abbreviations: BMI: Body mass index; DKK: Danish Krone. HbAA: Hemoglobin adducts of acrylamide; HbGA: Hemoglobin adducts of glycidamide; HbEO: Hemoglobin adducts of ethylene oxide; HOMA: homeostasis model assessment with HOMA IR: insulin resistance, HOMA β: pancreas beta cells activity, LDL: low density lipo-proteins; HDL: high density lipo-proteins.

Anthropometric measurements are based on clinical measurement at 20 years of age if available for the offspring or self-reported information when missing.

Large waist circumference indicates a waist circumference above 102 cm and 88 cm, respectively, for male and female offspring. Overweight or obese indicate a BMI > 25 kg/m^2^.

Metabolic syndrome was defined as having at least one ATP III criteria (waist circumference > 102 cm for males and > 88 cm for females; HDL cholesterol < 1.04 mmol/L for males and < 1.30 mmol/L for females; triglycerides ≥ 1.7 mmol/L, systolic blood pressure ≥ 130 mmHg or diastolic blood pressure ≥ 85 mmHg, or fasting blood sugar ≥ 6.1 mmol/L).

At age 20, 116 (18.2 %) of the 648 offspring were overweight or obese (BMI ≥ 25 kg/m^2^), and 140 (35.0 %) fulfilled at least one of the ATP III metabolic syndrome criteria (Beilby, 2004) . Mother and offspring’s characteristics were similar between the full study population with anthropometric measures and the subpopulation with metabolic biomarkers measures. The correlations between the adducts and the metabolic outcomes are presented in Supplemental Material Fig. S4.

Women excluded from analyses due to missing information on offspring at follow-up or missing adduct levels, had lower education, smoked more during pregnancy, and had higher mean levels of HbAA (132.1 ± 77.4 vs. 108.7 ± 68.8 pmol/g Hb) and HbGA (196.2 ± 120.9 vs. 166.3 ± 111.3 pmol/g Hb). Characteristics of the excluded offspring with missing adduct levels were similar to those of the included ones (Supplemental Material, Table S1).

### 3.2 Prenatal exposure to acrylamide

The mean level of HbAA was 108.7 ± 68.8 pmol/g Hb among the 638 included women, with smoking women having substantially higher levels as compared to non-smoking women (155.4 ± 82.5 vs. 83.3 ± 42.3 pmol/g Hb, Table 2). Similarly, the mean levels of HbGA and HbEO were also higher among smokers as expected (Table 2, Supplemental Material Table S2, Fig. S3). The Pearson correlation coefficient was 0.75 between HbAA and HbGA levels, and 0.71 between HbAA and HbEO levels. The distributions of HbAA, HbGA and HbEO were right-skewed (Supplemental Material, Fig. S3).

**Table 2:** Distribution of hemoglobin adduct levels (pmol/g) for the full study population and by maternal smoking status.

		n	missing	Mean ± SD	Min	P5	P25	Median (IQR)	P75	P95	Max
**Anthropometric outcome**											
HbAA											
	All	638	0	108.7 ± 68.8	27	43	63	85 (72.8)	135.8	238.2	650
	Non-smokers	413	0	83.3 ± 42.2	27	41	57	74 (37)	94	162.8	429
	Smokers	225	0	155.4 ± 82.5	39	58	94	138 (104)	198	302.8	650

HbGA											
	All	241	397	166.3 ± 111.3	26	53	82	138 (130)	212	376	647
	Non-smokers	101	312	101.6 ± 71.6	26	44	66	83 (45)	111	233	600
	Smokers	140	85	213.1 ± 111.5	50	73.65	138	187 (129.3)	267.3	444.2	647

HbEO											
	All	638	0	136.1 ± 137.7	20	34.85	56	86 (101.5)	157.5	418.3	1146
	Non-smokers	413	0	82.6 ± 76.5	23	33	49	67 (42.2)	91.2	168.4	851
	Smokers	225	0	234.3 ± 168.0	20	55	101	190 (213)	314	534.8	1146

**Metabolic biomarkers**											
HbAA											
	All	400	0	105.7 ± 61.1	33	43	62	82.5 (73)	135	228.25	405
	Non-smokers	255	0	80.9 ± 36.5	33	40	57	72 (35.5)	92.5	160.3	217
	Smokers	145	0	149.3 ± 70.6	39	57.2	94	137 (103)	197	271.4	405

HbGA											
	All	148	252	164.9 ± 110.2	26	53.35	80.75	137.5 (122)	202.8	374.3	647
	Non-smokers	57	198	95.3 ± 50.0	26	45	68	80 (30)	98	203.2	274
	Smokers	91	54	208.5 ± 115.2	50	67.5	132	181 (118.5)	250.5	446	647

HbEO											
	All	400	0	133.9 ± 136.0	20	34.95	56	84.46 (91.3)	147.3	418.1	874
	Non-smokers	255	0	77.4 ± 62.6	23	33.7	49	67 (40)	89	136.3	851
	Smokers	145	0	233.4 ± 169.5	20	55.2	99	183 (215)	314	579.8	874

HbAA: Hemoglobin adducts of acrylamide; HbGA: Hemoglobin adducts of glycidamide; HbEO: Hemoglobin adducts of ethylene oxide; SD: Standard deviation; IQR: Interquartile range.

Anthropometric outcomes include all offsprings with available measurements. Metabolic biomarkers include only a subsample of offsprings with metabolic biomarkers available.

### 3.3 Study population characteristics by quartiles of HbAA adduct levels and sex

In the study population with anthropometric measurements (N = 638), mothers with higher levels of HbAA tended to be younger, were more frequently of lower education, smoking, and of lower HEI scores (Supplemental Material, Table S3).

Supplemental Material, Table S4 summarizes the study population characteristics by offspring sex. Maternal characteristics were similar between female and male offspring. We did observe sex differences between female and male offspring. Female offspring had lower mean weight and height than males, but the BMI (22.2 ± 3.2 vs 22.8 ± 2.9 kg/m^2^) and number of overweight or obese offspring (59 (17.9 %) vs 57 (18.4 %)) were similar. Females had a lower mean blood sugar concentration, but a higher concentration of adiposity biomarkers as compared to males.

### 3.4 Associations between prenatal exposure to acrylamide and metabolic health at 20 years of age

For most of the outcomes examined within the full study population and after stratification by maternal smoking during pregnancy, there were no evidence of associations between prenatal exposure to AA and adult metabolic health (Table 3). No associations were evident for overweight or obesity, or metabolic syndrome in models fitted with HbAA on a continues scale (Table 3). Estimates were consistent for the full population and after restriction to offspring of non-smoking women, and for models with different degrees of adjustment (Supplemental Material, Table S5 and S6).

**Table 3:** Associations between prenatal exposure to acrylamide and metabolic health at 20 years of age.

	**All**		**Offspring of non-smokerss during pregnancy**	
	N	β (95 % CI)	N	β (95 % CI)
Outcomes on continuous scale				
Weight	638	−0.08 (−0.2, 0.08)	413	−0.04 (−0.3, 0.2)
Height	638	−0.07 (−0.2, 0.05)	413	−0.04 (−0.3, 0.2)
Waist circumference	638	0.06 (−0.07, 0.2)	413	0.05 (−0.2, 0.3)
Body mass index	638	−0.01 (−0.05, 0.03)	413	−0.004 (−0.07, 0.06)
Systolic blood pressure	400	−0.1 (−0.3, 0.09)	255	−0.2 (−0.5, 0.2)
Diastolic blood pressure	400	−0.02 (−0.2, 0.1)	255	−0.01 (−0.2, 0.2)
Triglycerides	400	−0.002 (−0.01, 0.007)	255	−0.01 (−0.03, 0.002)
Total cholesterol	400	0.008 (−0.009, 0.02)	255	−0.007 (−0.04, 0.02)
LDL cholesterol	400	0.01 (−0.002, 0.03)	255	0.002 (−0.02, 0.03)
HDL cholesterol	400	−0.003 (−0.01, 0.003)	255	−0.003 (−0.01, 0.008)
Blood sugar	400	0.004 (−0.005, 0.01)	255	0.01 (−0.003, 0.03)
Insulin*	400	0.36 (−0.51, 1.24)	255	0.84 (−0.75, 2.45)
Leptin*	400	1.10 (−1.30, 3.55)	254	1.21 (−3.00, 5.59)
Adiponectin*	400	0.11 (−0.70, 0.92)	255	1.12 (−0.29, 2.55)
HOMA IR index*	400	0.43 (−0.49, 1.36)	255	1.08 (−0.61, 2.79)
HOMA β Index*	400	0.58 (−0.62, 1.80)	255	1.18 (−1.16, 3.58)

Categorical outcomes	N	OR (95 % CI)	N	OR (95 % CI)
Overweight or Obese	638	0.98 (0.95, 1.02)	413	0.98 (0.91, 1.04)
Large waist	638	1.00 (0.96, 1.04)	413	0.99 (0.89, 1.07)
Metabolic syndrome	400	1.04 (0.99, 1.08)	255	1.08 (1.01, 1.17)

Abbreviations: CI; confidence intervals, HOMA: homeostasis model assessment with HOMA IR: insulin resistance, HOMA β: pancreas beta cells activity, LDL: low density lipo-proteins; HDL: high density lipo-proteins.

Average levels of each outcome or number of cases can be found in Table 1.

Overweight or Obese: body mass index ≥ 25 kg/m^2^. Large waist: Waist circumference above 102 or 88 cm for male or female. Metabolic syndrome was defined as having at least one ATP III criteria (waist circumference > 102 cm for males and > 88 cm for females; HDL cholesterol < 1.04 mmol/L for males and < 1.30 mmol/L for females; triglycerides ≥ 1.7 mmol/L, systolic blood pressure ≥ 130 mmHg or diastolic blood pressure ≥ 85 mmHg, or fasting blood sugar ≥ 6.1 mmol/L).

Estimates are expressed by 10 pmol/g Hb increase in hemoglobin adducts of acrylamide (HbAA) and adjusted for household income, maternal education, maternal age, maternal smoking, and maternal healthy eating index in pregnancy.

The models stratified by smoking status is restricted to non-smoking mothers, and adjusted for household income, maternal education, maternal age and maternal healthy eating index in pregnancy.

* Natural logarithm transformed outcome; estimates expressed as percentage of change (95% CI).

**Fig. 1:**
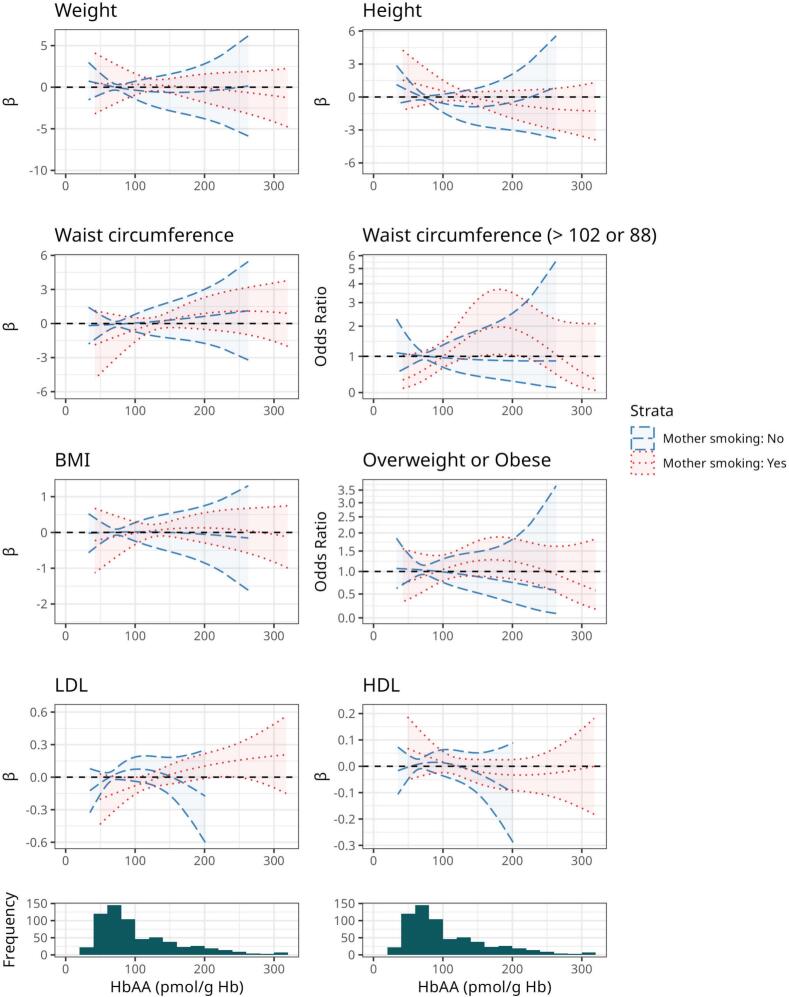
Dose-response curves of the associations between prenatal exposure to acrylamide and metabolic health at 20 years of age. The curves are expressed relative to minimum exposure with 2df (lowest AIC). They are adjusted for household income, maternal education, maternal age, maternal smoking, and maternal healthy eating index in pregnancy. The models stratified by smoking status are restricted to non-smoking mother or smoking mother offspring pairs and adjusted for household income, maternal education, maternal age, and maternal healthy eating index in pregnancy. The solid lines show the association for all participants, the dashed lines for non-smokers mothers and the dotted lines for smokers. BMI: Body mass index; HbAA: hemoglobin adducts from acrylamide; HDL: high density lipoprotein; LDL: low density lipoprotein.

Fig. 1 and S5 illustrate the shapes of the dose–response curves for the full study population and after stratification by maternal smoking status. These plots showed quasi linear patterns, with most of offspring having adducts levels under 250 pmol/g Hb, and the likelihood ratio tests against linear models were all non-significant except for waist circumference. The shapes and directions of the associations were similar in non-smokers and smokers.

### 3.5 Stratified and sensitivity analyses

**Fig. 2:**
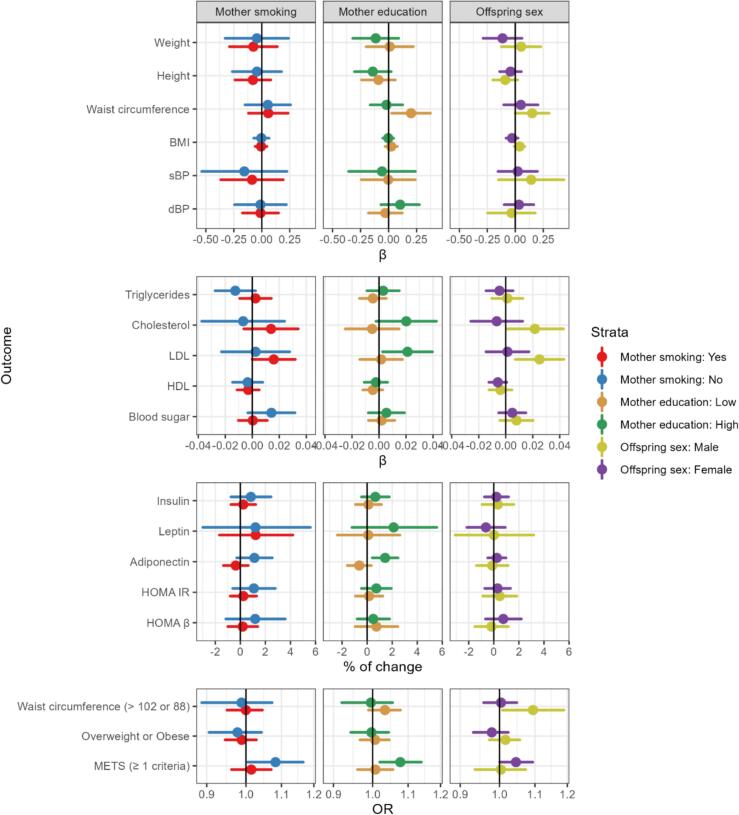
Associations between prenatal exposure to acrylamide and metabolic health at age 20 among stratified by maternal smoking status, education and sex, respectively. All the effect estimates are express per 10 pmol/g Hb increase of HbAA and adjusted for household income, maternal education, maternal age, maternal smoking, and maternal healthy eating index in pregnancy. Maternal smoking and education were removed from adjustment when models were stratified on these characteristics. OR: Odds ratio. Overweight or Obese: BMI ≥ 25 kg/m^2^. METS: metabolic syndrome defined as having at least one ATP III criteria (waist circumference > 102 cm for males and > 88 cm for females; HDL cholesterol < 1.04 mmol/L for males and < 1.30 mmol/L for females; triglycerides ≥ 1.7 mmol/L, systolic blood pressure ≥ 130 mmHg or diastolic blood pressure ≥ 85 mmHg, or fasting blood sugar ≥ 6.1 mmol/L).

For most of the associations, we also observed lack of evidence of associations consistently across the stratified analysis on maternal smoking status, offspring sex and maternal educational level (Fig. 2).

When stratifying analyses on maternal smoking the associations for LDL cholesterol (Table 2, Fig. 1) were strongest among offspring of smokers and null in offspring of non-smokers (Fig. 2). In offspring of non-smokers, HbAA levels were associated with metabolic syndrome as well as higher levels of blood sugar and adiponectin (Fig. 2). We consistently observed an increase in waist circumference and LDL cholesterol with higher levels of AA across different analyses and strata. The mean (95 % CI) increases of waist circumference corresponded to 0.06 (−0.07, 0.2) cm and 0.05 (−0.2, 0.3) cm per 10 pmol/g Hb increase in HbAA levels in the full study population and the non-smoking population, respectfully, after adjustment (Table 3). Offsprings in the higher quartile of exposure had on average 2 (−0.4, 4) cm larger waist circumference (Table S7). This association was stronger in males than females (interaction p-value < 0.001, Fig. 2). For LDL cholesterol, the average increase was 0.01 (−0.002, 0.03) mmol/L, while smaller with restriction to offspring of non-smokers. LDL cholesterol levels of offspring in the highest quartile of exposure were 0.2 (0.01, 0.5) mmol/L higher compared to the lowest exposure quartile. This association was also stronger in males than in females (interaction p-value = 0.016, Fig. 2).

Similar patterns were observed for HbGA with even stronger associations with LDL cholesterol and HDL concentrations (Supplemental Material, Table S6). Although, stratified analyses suggested that associations for waist circumference, LDL and total cholesterol were evident in males and not in females (Fig. 2), most of results observed in the full population (Table 2) were almost identical to those from models fitted with sex-specific z-score instead of crude metabolic outcomes (Supplemental Material, Table S8).

The results for the entire population and for the stratified analysis were essentially unchanged when including only complete cases and when including offspring with and without clinical examination anthropometrics measurement (Supplemental Material Table S9). Supplemental Material Table S11 Using the sum of HbAA and HbGA as the exposure revealed similar results to those presented for models fitting HbAA and HbGA adduct levels one-by-one in separate models (Supplemental Material Table S10). The Supplemental Material Table S11 summarize the results of the multiple linear regression as standarized effect estimates. Finally, when stratifying the analyses according to maternal level of education, the association between higher HbAA levels and larger waist circumference was only evident among offspring of mothers of low education while the association for LDL cholesterol was only evident among offspring whose mother had a high education (Supplemental Material Table S12).

## 4 Discussion

In this biomarker-based birth cohort study we observe a substantial variation in prenatal exposure to AA, without clinically relevant associations with measures of metabolic health in the offspring at age 20 years. We found a tendency toward a higher waist circumference and higher levels of LDL cholesterol by higher prenatal exposure to AA in adult offspring across all models and subgroups, in particular among male offspring, although not consistently statistically significant. Associations with other metabolic health outcomes were mostly null or close to null and only statistically significant in various subgroups limiting the confidence in the observations.

Indications that maternal exposure to AA from diet during pregnancy may affect offspring metabolic health have, so far, been provided by a single study relying on self-reported maternal recalls of dietary intakes during pregnancy and offspring measures of height and weight at 3, 5 and 8 years of age in children born in Norway in 1998–2008 (Kadawathagedara et al. 2018). Another study based on estimated AA intake from diet in 6,022 adults reported that the estimated AA was associated with increased incidence of type 2 diabetes in females in a cohort study from Iran, but not in males (Hosseini-Esfahani et al. 2023).

A growing and large (n ∼ 100) number of publications have evaluated human exposure to AA by analyses of HbAA levels (Pedersen et al. 2022), but almost all of these are based on exposures during adulthood and only few studies have evaluated prenatal exposure (Chandia-Poblete et al., 2025, Hogervorst et al., 2021, Pedersen et al., 2012, Schettgen et al., 2004, Von Stedingk et al., 2011) variation in the adduct levels not only because of exposure to tobacco smoke, but also among infants of non-smokers, suggesting that prenatal exposure to AA can be modified by dietary changes. Measured Hb adducts levels from AA, GA and EO in blood obtained from pregnant women or their newborns, were concluded to reflect the same “area under the concentration–time curve” of the compounds in the mother and newborn, respectively, during the third trimester (Von Stedingk et al. 2011). Further, recently it was published that AA could be detected as the short-lived, free compound present in amniotic fluid from the second trimester (Vrachnis et al. 2023).

Previous studies based on HbAA have focused on metabolic health in non-pregnant adults and have reported mixed evidence that AA is associated with poor metabolic health. Among the multiple cross-sectional studies with non-pregnant adults based on the US National Health and Nutrition Examination Survey (NHANES) with evidence supportive of association between HbAA and metabolic health outcomes is limited to a single study, in which the mean serum concentration of triglyceride was found to be highest among the individuals with the highest quartile of HbAA, but there were no associations between HbAA with total cholesterol, LDL or HDL concentrations (Cheang et al., 2021, Hung et al., 2021). Other NHANES studies with non-pregnant adults have reported null or even inverse associations between HbAA levels and metabolic outcomes like body weight (Chu et al. 2017), BMI (Vesper et al. 2013), waist circumference, subscapular/triceps skinfold, body fat parameters (Chu et al. 2017), blood glucose (Hung et al. 2021), prevalence of obesity (Huang et al., 2018, Yin et al., 2022), abdominal obesity (Huang et al., 2018, Hung et al., 2021, Yin et al., 2022), overweight (Huang et al. 2018), diabetes mellitus (Yin et al. 2022), and metabolic syndrome (Wan et al. 2022). Furthermore, higher HbAA levels were not found to be associated with higher prevalence of hypertension or blood pressure in a NHANES study of adolescents aged 13–19 years (Liang et al. 2022). Additional cross-sectional studies with HbAA measured in adults from Europe have reported inverse associations between HbAA and BMI (Vesper et al. 2008). HbAA adduct levels have also been reported not to be correlated with the body weight of adults from Norway (Bjellaas et al. 2007). Notable, in many of these studies results are inconsistent across HbAA, HbGA, the HbGA/HbAA ratio, and subgroups. For instance, while higher HbAA was associated with lower BMI, higher HbGA and HbAA/HbGA levels were associated with higher BMI in non-smokers (Vesper et al. 2008).

In our study, HbAA and HbGA showed consistent results, both in the entire population and in the stratified analyses. The point estimates were slightly higher for HbGA as compared with those of HbAA. This is likely due to bias resulting from the higher limit of detection of HbGA excluding offspring of non-smoking women who had lower adducts levels. This is supported by the similarity between effect estimates and the shape of the dose–response curves of HbGA and of HbAA results among offspring of women who smoked during pregnancy.

Both AA and metabolic health are associated with dietary and smoking habits. As expected, in the current study population we observed that maternal HbAA levels were associated with both smoking and consumption of unhealthy diet and that maternal smoking was associated with a higher score of unhealthy diet. Epidemiological studies on health effects of AA are complementary to experimental studies as exposure to AA in humans does not occur in isolation from other factors such as dietary habits which can act together with AA.

Like in previous studies of prenatal exposure to AA (Chandia-Poblete et al., 2025, Hogervorst et al., 2022), maternal smoking during pregnancy causes lower birth weight and the underlying biological mechanisms involved may be partly shared between AA and other components of tobacco smoke. Tobacco smoking is a main source of exposure to AA in addition to particles and multiple other toxicants such as polycyclic aromatic hydrocarbons, nicotine, cadmium that can induce hypoxia and reduce placental blood exchange resulting in prenatal growth restriction and disrupt human development in early life (Rogers 2019). Women who smoke during pregnancy are also likely be characterized by unhealthy habits such as a higher intake of diet rich in AA.

Most of the associations evaluated in our study were similar in offspring of non-smokers and smokers, but not all (Fig. 1, Fig. 2). As such, the associations for LDL cholesterol were strongest among offspring of smokers and reduced to null in offspring of non-smoking women. However, in offspring of non-smokers, we observed that HbAA levels were associated with metabolic syndrome as well as higher levels of blood sugar and adiponectin.

Despite the health concern that has been raised since the discovery of the formation of AA in foods two decades ago, focus on prenatal exposure as a critical window of exposure with heighten vulnerability towards exposure to toxicants like AA, has been understudied in terms of long-lasting health effects. Our study is motivated by the developmental origin of health and disease concept which was first proposed as fetal origins of diseases in the 1990 s from studies on birth weight and cardiovascular disease (Barker 1999). This has now advanced to a broader concept where it is evident that most non-communicable diseases may partly have their origin in early life, including metabolic and cardiovascular disorders and that multiple factors early in life can have immediate and long-term effects on health (Poston et al. 2022). Although these disorders are a leading cause of death worldwide, the role of exposure to chemicals such as AA throughout different periods of life in metabolism, growth, and reproduction, is not fully clarified (Matoso et al. 2019). In the same study population, we have observed that higher HbAA levels were associated with higher odds ratio of clinician-based fetal growth restriction (FGR), and small-for-gestational age (SGA) and reduction in neonatal size at birth in terms of birth weight, length and head circumference in the whole cohort, before and after adjustment for smoking, particularly among the highest exposed mother–child pairs, and after restriction to offspring of smokers (Chandia-Poblete et al. 2025). Our findings of associations with clinician-based FGR strengthen existing evidence that higher levels of prenatal exposure to AA reduce the fetal growth and highlights the need to increase the efforts to reduce maternal AA exposure from both diet and smoking during pregnancy. It is biologically plausible that the prenatal exposure to AA from maternal diet and other sources not only restrict fetal growth, but also disturb metabolic health of the offspring (Buyukdere and Akyol 2023). However, according to our results, these exposures seemed not to have lasting influence on early adult metabolic health. After ingestion, AA is easily absorbed and distributed in the body to different organs (Calleman, 1996, European Food Safety Agency [EFSA], Panel on Contaminants in the Food Chain (CONTAM), 2015). AA is metabolized by the mercapturic acid pathway and to the more reactive epoxide glycidamide (GA) by cytochrome P450 2E1 enzyme in the liver (Sumner et al. 1999). Free AA is rapidly excreted in the urine by kidneys and the *in vivo* half-life of free AA in humans has been estimated to a few hours (Fennell et al., 2005, Fuhr et al., 2006). Prenatal exposure to AA is of concern as AA crosses the human placenta (Annola et al., 2008, Sörgel et al., 2002). AA, as well as its metabolite GA, are highly reactive compounds that in blood can be detected in the form of stable covalent adducts of Hb, HbAA, and HbGA, respectively (Friedman, 2003, Pedersen et al., 2022, Törnqvist et al., 2002, Törnqvist, 2005). These adducts reflect exposure to AA over the past four months (Törnqvist 2005).

A notable strength of our study was that the exposure assessment is based on measurement of HbAA which reflects cumulative exposure and uptake through all routes of exposure (Pedersen et al. 2022). For this study, we measured the Hb adducts in maternal blood collected in 30 week’ gestation. This represents an exposure during an earlier and longer window of the critical prenatal development than e.g. measures in umbilical cord blood which represent exposures during a time closer to birth. One advantage of using maternal blood relates to certainty of the lifespan of the erythrocytes which is less certain in cord blood. Since maternal blood has the double amount of N-terminal valines on Hb compared to cord blood, relatively higher levels of adducts from reactive electrophiles can be formed and hereby detectability of Hb adducts in maternal blood is improved. We have previously observed that the cord blood HbAA levels were approximately half of those measured in the corresponding mothers and that the correlation between maternal and cord blood Hb adducts levels was high (Pedersen et al., 2012, Von Stedingk et al., 2011). The quantification of HbGA adduct levels with the Fire method has proved to be more challenging than the quantification of HbAA, with a higher degree of missing observations in non-smoking women. The truncation of the HbGA levels range is a limitation as we could not report effect estimates for the population with the lowest exposure subset and the exclusion of participants with lower adduct levels may explain the increased estimates in HbGA models compared to HbAA models.

Another strength was that we had access to a wide range of anthropometric and biological measurements rather than solely offspring recalls or parental recalls in early childhood and hereby bias related to recall bias or measurement errors is reduced.

A third strength was the substantially longer duration of follow-up of 20 years, compared to previous Norwegian study with follow-up of 8 years (Kadawathagedara et al. 2018). Measurements of metabolic health in early adulthood allow for early management of metabolic dysfunction but might be too early for detection of metabolic or cardiovascular disease, like type 2 diabetes or heart failure. We cannot rule out that the metabolic health of the offspring at this age, at younger or older age were influenced by unmeasured factors that influence both the prenatal exposure to AA and offspring metabolism such as changes in maternal diet, environmental exposures or genes. We did not adjust for postnatal offspring factor (i.e. lifestyle, physical activity, diet, sleep) as they occur after the exposure and cannot affect prenatal exposure to AA. However, they may well be mediators of the associations between AA and metabolic health.

Other strengths of our study are related to the adjustment for smoking and socio-economic status which reduce the potential confounding and allow us to stratify by smoking status. Moreover, we were able to analyze a large diversity of metabolic health outcome and include extensive stratified and sensitivity analysis.

The blood of the current study was collected in 1988. This enables a long follow-up period of the offspring. The blood samples were stored at −20℃ for approximately 35 years before analysis. There is no evidence or any indication from previous work to support that measures of HbAA adduct levels change over time by long storage of blood at −20℃. Even if unlikely, it cannot be absolutely ruled out that hypothetically a long storage period could have a small influence on measured levels. However, in the present study all the blood samples have been stored under the same conditions and during the same time, and therefore differential exposure misclassification related to this long storage duration is minimized.

The Danish population of the current study was relatively homogenous, well-resourced and with relatively high levels of education and cohort participants are often of higher socioeconomic status and healthier than the general population. Thus, the findings from this study might not be valid in populations with greater diversity in educational levels, health and resources. However, we observed no difference in the prevalence of overweight and obesity of the included and excluded population for which we had data to compare and in between the two populations. The mean age, BMI, smoking and dietary habits of the women in our study differed from those of women who are pregnant now in Denmark. In general, pregnant women in high-income countries, are slightly older, have higher BMI and smoke less during pregnancy nowadays. The diet has also changed towards a higher intake of more fast-paced, ready-made, ultra processed and processed food including more food prepared at high-temperature such as potato chips, pizza, white breads and pastries. For instance, boiled potatoes are less commonly consumed in Denmark nowadays as compared with the late 1980 s. The volume of salty chips and sweets including chocolate that can have high concentrations of AA has increased as we become richer, and the type of coffee consumed has changed towards higher intake of espresso and lower intake of filtered coffee. Despite these changes in diet over time, the variation and the median HbAA levels were relatively high compared to levels reported in cord blood (Pedersen et al. 2012), also among non-smokers in the current study. However, the analyses are not intercalibrated and the HbAA levels are not directly comparable across different studies (Pedersen et al. 2022).

The prevalence of young adults with overweight has also increased over time to 56.3 % and 39.6 % in men and women, respectively, in 2010 in Denmark (Toft et al. 2015). Although, the findings from this study might not be generalizable for the current population, these differences do not influence the internal validity of our study.

Pregnancy and early life periods both present a unique opportunity to protect lifelong health and wellbeing. However apart from the consistent evidence of prenatal exposure to AA with lower birth weight (Chandia-Poblete et al., 2025, Hogervorst et al., 2022), very little is known about the potential impact of prenatal exposure to AA on human health. More longitudinal studies, using accurate Hb biomarker measures, are encouraged to elucidate all the potential effects of AA on human health.

Overall, there was little evidence that prenatal exposure to AA was associated with metabolic outcomes measured at age 20. We evaluate associations between prenatal exposure to AA and multiple outcomes to capture metabolic health more broadly. However, this may result in chance findings. Thus, the findings of associations between prenatal exposure to AA and higher levels of LDL cholesterol and waist circumference warrant replication as well as the increased risk of metabolic syndrome observed in offspring of non-smokers as it is biologically plausible that early life exposure to AA may increase the risk of poor metabolic health.

## CRediT authorship contribution statement

**Stéphane Tuffier:** Writing – review & editing, Writing – original draft, Visualization, Methodology, Investigation, Formal analysis, Data curation. **Efstathios Vryonidis:** Writing – review & editing, Methodology, Investigation, Data curation. **Thorhallur Ingi Halldorsson:** Writing – review & editing, Methodology, Conceptualization. **Anne Ahrendt Bjerregaard:** Writing – review & editing, Methodology, Conceptualization. **Damian Chandia-Poblete:** Writing – review & editing, Methodology. **Dorte Rytter:** Writing – review & editing, Methodology, Conceptualization. **Bodil Hammer Bech:** Writing – review & editing, Methodology, Conceptualization. **Tine Brink Henriksen:** Writing – review & editing, Methodology, Conceptualization. **Thorkild I.A. Sørensen:** Writing – review & editing, Methodology, Conceptualization. **Sjurdur Frodi Olsen:** Writing – review & editing, Resources, Project administration, Methodology, Investigation, Data curation, Conceptualization. **Margareta Törnqvist:** Writing – review & editing, Resources, Methodology, Investigation, Data curation. **Marie Pedersen:** Writing – review & editing, Supervision, Resources, Project administration, Methodology, Investigation, Funding acquisition, Conceptualization.

## Funding

This study is part of the CHIPS project which has received funding from the European Research Council (ERC) under the European Union’s Horizon 2020 research and innovation program (grant agreement No. 758151).

## Declaration of competing interest

The authors declare that they have no known competing financial interests or personal relationships that could have appeared to influence the work reported in this paper.

## Data Availability

The authors do not have permission to share data.
