# Supplementary material for: Prenatal exposure to acrylamide and metabolic health at 20 years of age A biomarker-based Danish cohort study
Source: Environ Int. 2026 Jan;207:110000. doi: 10.1016/j.envint.2025.110000 (PMC12819368; doi:10.1016/j.envint.2025.110000)
Supplement: Supplementary Data 1 [file mmc1.docx]

Supplemental Material

A Biomarker-based Cohort Study from Denmark of Prenatal Exposure to Acrylamide and Metabolic Status at 20 Years of Age

Stéphane Tuffier, Efstathios Vryonidis, Thorhallur Ingi Halldorsson, Anne Ahrendt Bjerregaard, Damian Chandia-Poblete, Dorte Rytter, Bodil Hammer Bech, Tine Brink Henriksen, Thorkild I.A. Sørensen, Sjurdur Frodi Olsen, Margareta Törnqvist, Marie Pedersen.

# Methods

### Healthy eating index (HEI)

Healthy eating index was calculated from the mother food frequency questionnaires. From the questionnaires, daily consumption and quantity of fruits and vegetables, fiber, fish, meat, salt (sodium), added sugar or saturated fatty acid as well as total daily energy were estimated. Estimations with abnormal energy intake levels were removed (zero, <2,500 or >25,000 KJ/days). Each category was weighted from 0 (worst) to 10 (best) points according to the Danish and Nordic nutritional guidelines (Blomhoff et al. 2023; Ministry of Environment and Food of Denmark). HEI is then defined as the sum of the eight scores and range from 0 to 80.

| Category | Best (10pts) | Worst (0pts) |
| --- | --- | --- |
| Fruits and veg^1^ | 600 g/d | 0 |
| Fiber | 30 g/d | 0 |
| Fish | 350 g/d | 0 |
| Meat^2^ | 200 g/d | 500 g/d |
| Saturated fatty acid^2^ | 0% of total energy | 10% of total energy |
| Sodium^2^ | 1.6 g/d | 2.5 g/d |
| Soft drinks^2, 3^ | 0 g/d | 500 g/d |
| Added sugar^2^ | 0% of total energy | 10% of total energy |
| 1. Divided by 2 if fruit quantity was superior to vegetables.  2. Inversed score, with 10 points for the lowest quantity  3. 250g for juice, gaseous water, cacao drinks and 350g for soda  g/d: gram per day; pts: point | | |

Blomhoff R, Andersen R, Arnesen EK, Christensen JJ, Eneroth H, Erkkola M, et al. 2023. *Nordic Nutrition Recommendations 2023 : Integrating Environmental Aspects*. Nordisk Ministerråd.

Ministry of Environment and Food of Denmark. De officielle kostråd (Officiel Danish dietary guidelines). Available: https://foedevarestyrelsen.dk/kost-og-foedevarer/alt-om-mad/de-officielle-kostraad [accessed 8 July 2024].

# Table S*1*: Characteristics of the excluded and included study population.

|  | Excluded (n=353) | | Included (n=638) | |  |
| --- | --- | --- | --- | --- | --- |
|  | N (missing) |  | N (missing) |  | p-value |
| Maternal characteristics |  |  |  |  |  |
| Maternal age (years), mean ± SD | 353 (0) | 29 ± 4.6 | 638 (0) | 29.2 ± 4.1 | 0.61 |
| Length of education, n (%) |  |  |  |  | <0.001 |
| Other or no education |  | 37 (10.5) | 60 | 60 (9.4) |  |
| Short education |  | 163 (46.2) | 211 | 211 (33.1) |  |
| Intermediate education |  | 113 (32) | 260 | 260 (40.8) |  |
| Academic education |  | 40 (11.3) | 107 | 107 (16.8) |  |
| Household income (DKK), n (%) |  |  |  |  | 0.005 |
| <150,000 DKK |  | 58 (16.4) | 104 | 104 (16.3) |  |
| 150,000 to 200,000 DKK |  | 97 (27.5) | 115 | 115 (18) |  |
| 200,000 to 300,000 DKK |  | 101 (28.6) | 212 | 212 (33.2) |  |
| > 300,000 DKK |  | 97 (27.5) | 207 | 207 (32.4) |  |
| Healthy eating index, mean ± SD | 337 (16) | 35.1 ± 7.1 | 638 (0) | 34.6 ± 6.8 | 0.33 |
| Smoking pregnancy, n (%) | 149 (0) | 149 (42.2) | 225 (0) | 225 (35.3) | <0.001 |
| HbAA (pmol/g), mean ± SD | 261 (92) | 132.1 ± 77.4 | 638 (0) | 108.7 ± 68.8 | <0.001 |
| HbGA (pmol/g), mean ± SD | 129 (224) | 196.2 ± 120.9 | 241 (397) | 166.3 ± 111.3 | 0.021 |
| Pre-pregnancy body mass index (kg/m2), mean ± SD | 353 (0) | 21.7 ± 3.6 | 638 (0) | 21.3 ± 2.9 | 0.089 |
| Overweight or Obese, n (%) | 38 (0) | 38 (10.8) | 50 (0) | 50 (7.8) | 0.20 |
| Offspring’s characteristics |  |  |  |  |  |
| Offspring sex, n (%) |  |  |  |  | 0.14 |
| Male |  | 23 (37.7) | 309 | 309 (48.4) |  |
| Female |  | 38 (62.3) | 329 | 329 (51.6) |  |
| Weight (kg), mean ± SD | 60 (293) | 66.1 ± 13.2 | 638 (0) | 69.8 ± 12.4 | 0.040 |
| Height (cm), mean ± SD | 60 (293) | 173.1 ± 9.1 | 638 (0) | 175.8 ± 9.5 | 0.029 |
| Waist circumference (cm), mean ± SD | 60 (293) | 80.2 ± 9.7 | 638 (0) | 82.1 ± 9.5 | 0.15 |
| Body mass index (kg/m^2^), mean ± SD | 60 (293) | 21.9 ± 3.5 | 638 (0) | 22.5 ± 3.1 | 0.25 |
| Overweight or Obese, n (%) | 8 (293) | 8 (13.3) | 116 (0) | 116 (18.2) | <0.001 |
| Systolic blood pressure (mmHg), mean ± SD | 42 (311) | 109.9 ± 10.1 | 401 (237) | 110.7 ± 10.9 | 0.63 |
| Diastolic blood pressure (mmHg), mean ± SD | 42 (311) | 66.3 ± 7.2 | 401 (237) | 65.9 ± 6.6 | 0.72 |
| Triglycerides (mmol/L), mean ± SD | 41 (312) | 1 ± 0.4 | 400 (238) | 1 ± 0.4 | 0.54 |
| Total cholesterol (mmol/L), mean ± SD | 41 (312) | 4.5 ± 0.9 | 400 (238) | 4.4 ± 0.9 | 0.60 |
| LDL cholesterol (mmol/L), mean ± SD | 41 (312) | 2.5 ± 0.8 | 400 (238) | 2.5 ± 0.7 | 0.76 |
| HDL cholesterol (mmol/L), mean ± SD | 41 (312) | 1.5 ± 0.3 | 400 (238) | 1.5 ± 0.3 | 0.54 |
| Blood sugar measured with Accu-Chek (mmol/L), mean ± SD | 42 (311) | 4.9 ± 0.4 | 401 (237) | 4.9 ± 0.5 | 0.65 |
| Insulin (pmol/L), mean ± SD | 41 (312) | 43.3 ± 15.9 | 400 (238) | 43.5 ± 21.6 | 0.92 |
| Leptin (μg/L), mean ± SD | 41 (312) | 12 ± 11.9 | 400 (238) | 11.8 ± 12.4 | 0.93 |
| Adiponectin (mg/L), mean ± SD | 41 (312) | 8.3 ± 3.3 | 400 (238) | 8.9 ± 3.7 | 0.25 |
| HOMA IR index, mean ± SD | 41 (312) | 1.4 ± 0.5 | 400 (238) | 1.4 ± 0.8 | 0.65 |
| HOMA β Index, mean ± SD | 41 (312) | 97.2 ± 47 | 400 (238) | 95.4 ± 62.7 | 0.83 |
| Metabolic syndrome (one criteria or more), n (%) | 14 (312) | 14 (34.1) | 140 (238) | 140 (35) | <0.001 |

Abbreviations: BMI: Body mass index; DKK: Danish Krone. HbAA: Hemoglobin adducts of acrylamide; HbGA: Hemoglobin adducts glycidamide; HbEO: Hemoglobin adducts ethylene oxide; HOMA: homeostasis model assessment with HOMA IR: insulin resistance, HOMA β: pancreas beta cells activity, LDL: low density lipo-proteins; HDL: high density lipo-proteins.

Anthropometric measurements are based on clinical measurement at 20 years of age if available for the offspring or self-reported information when missing. Large waist circumference indicates a waist circumference above 102 cm and 88 cm, respectively, for male and female offspring. Metabolic syndrome was defined as having at least one ATP III criteria (waist circumference > 102 cm for males and > 88 cm for females; HDL cholesterol < 1.04 mmol/L for males and < 1.30 mmol/L for females; triglycerides ≥ 1.7 mmol/L, systolic blood pressure ≥ 130 mmHg or diastolic blood pressure ≥ 85 mmHg, or fasting blood sugar ≥ 6.1 mmol/L).

# ****Table S2********: Study population characteristics by**** ****maternal smoking status during pregnancy.****

|  | Anthropometric outcomes | | | | Metabolic biomarkers | | | |
| --- | --- | --- | --- | --- | --- | --- | --- | --- |
|  | **All (n=638)** | **Maternal Smoking** | | | **All (n=400)** | **Maternal Smoking** | | |
|  |  | **No (n=413)** | **Yes (n=225)** | **p-value** |  | **No (n=255)** | **Yes (n=145)** | **p-value** |
| Maternal characteristics |  |  |  |  |  |  |  |  |
| Maternal age (years), mean ± SD | 29.2 ± 4.1 | 29.4 ± 4.1 | 28.8 ± 4 | 0.07 | 29.2 ± 4.1 | 29.5 ± 4.2 | 28.7 ± 3.8 | 0.049 |
| Length of education, n (%) |  |  |  | <0.001 |  |  |  | <0.001 |
| Other or no education | 60 (9.4) | 37 (9) | 23 (10.2) |  | 41 (10.2) | 23 (9) | 18 (12.4) |  |
| Short education | 211 (33.1) | 110 (26.6) | 101 (44.9) |  | 121 (30.2) | 59 (23.1) | 62 (42.8) |  |
| Intermediate education | 260 (40.8) | 193 (46.7) | 67 (29.8) |  | 167 (41.8) | 123 (48.2) | 44 (30.3) |  |
| Academic education | 107 (16.8) | 73 (17.7) | 34 (15.1) |  | 71 (17.8) | 50 (19.6) | 21 (14.5) |  |
| Household income (DKK), n (%) |  |  |  | 0.24 |  |  |  | 0.23 |
| <150,000 | 104 (16.3) | 67 (16.2) | 37 (16.4) |  | 66 (16.5) | 43 (16.9) | 23 (15.9) |  |
| 150,000 to 200,000 | 115 (18) | 72 (17.4) | 43 (19.1) |  | 67 (16.8) | 44 (17.3) | 23 (15.9) |  |
| 200,000 to 300,000 | 212 (33.2) | 129 (31.2) | 83 (36.9) |  | 135 (33.8) | 77 (30.2) | 58 (40) |  |
| > 300,000 | 207 (32.4) | 145 (35.1) | 62 (27.6) |  | 132 (33) | 91 (35.7) | 41 (28.3) |  |
| Healthy eating index, mean ± SD | 34.6 ± 6.8 | 35.1 ± 6.6 | 33.7 ± 7.1 | 0.011 | 34.6 ± 6.8 | 35.1 ± 6.5 | 33.8 ± 7.2 | 0.067 |
| Pre-pregnancy BMI (kg/m^2^), mean ± SD | 21.3 ± 2.9 | 21.4 ± 3 | 21.2 ± 2.6 | 0.43 | 21.4 ± 3 | 21.5 ± 3.1 | 21.3 ± 2.8 | 0.56 |
| Overweight or Obese, n (%) | 50 (7.8) | 35 (8.5) | 15 (6.7) | 0.005 | 32 (8) | 22 (8.6) | 10 (6.9) | 0.034 |
| Smoking pregnancy, n (%) | 225 (35.3) |  |  |  | 145 (36.3) |  |  |  |
| HbAA (pmol/g Hb), mean ± SD | 108.7 ± 68.8 | 83.3 ± 42.3 | 155.4 ± 82.5 | < 2e-16 | 105.7 ± 61.1 | 80.9 ± 36.5 | 149.3 ± 70.6 | < 2e-16 |
| HbGA (pmol/g Hb), mean ± SD | 166.3 ± 111.3 | 101.6 ± 71.6 | 213.1 ± 111.5 | < 2e-16 | 164.9 ± 110.2 | 95.3 ± 50.0 | 208.5 ± 115.3 | 1.65e-13 |
| HbEO (pmol/g Hb), mean ± SD | 136.1 ± 137.7 | 82.6 ± 76.5 | 234.3 ± 168.0 | < 2e-16 | 133.9 ± 136.0 | 77.4 ± 62.6 | 233.4 ± 169.5 | < 2e-16 |
| Offspring’s characteristics |  |  |  |  |  |  |  |  |
| Sex, n (%) |  |  |  | 0.36 |  |  |  | 0.37 |
| Female | 309 (48.4) | 206 (49.9) | 103 (45.8) |  | 162 (40.5) | 108 (42.4) | 54 (37.2) |  |
| Male | 329 (51.6) | 207 (50.1) | 122 (54.2) |  | 238 (59.5) | 147 (57.6) | 91 (62.8) |  |
| Weight (kg), mean ± SD | 69.8 ± 12.4 | 69.6 ± 12 | 70 ± 13.1 | 0.75 | 68.4 ± 12.5 | 68.2 ± 12.2 | 68.8 ± 13.1 | 0.63 |
| Height (cm), mean ± SD | 175.8 ± 9.5 | 176.4 ± 9.4 | 174.8 ± 9.8 | 0.048 | 174.2 ± 9.2 | 174.7 ± 9 | 173.4 ± 9.4 | 0.16 |
| Waist circumference (cm), mean ± SD | 82.1 ± 9.5 | 81.4 ± 8.7 | 83.3 ± 10.8 | 0.022 | 81.3 ± 8.6 | 80.8 ± 8.3 | 82.1 ± 9.2 | 0.16 |
| Large waist circumference, n (%) | 62 (9.7) | 29 (7) | 33 (14.7) | 0.61 | 37 (9.2) | 18 (7.1) | 19 (13.1) | 0.87 |
| BMI (kg/m^2^), mean ± SD | 22.5 ± 3.1 | 22.3 ± 2.9 | 22.8 ± 3.3 | 0.052 | 22.4 ± 3.1 | 22.2 ± 3 | 22.8 ± 3.4 | 0.096 |
| Overweight or Obese, n (%) | 116 (18.2) | 66 (16) | 50 (22.2) | 0.14 | 73 (18.2) | 38 (14.9) | 35 (24.1) | 0.73 |
| Systolic blood pressure (mmHg), mean ± SD |  |  |  |  | 110.7 ± 10.9 | 110.3 ± 10.7 | 111.4 ± 11.2 | 0.33 |
| Diastolic blood pressure (mmHg), mean ± SD |  |  |  |  | 65.9 ± 6.6 | 65.4 ± 6.6 | 66.7 ± 6.4 | 0.06 |
| Triglycerides (mmol/L), mean ± SD |  |  |  |  | 1 ± 0.4 | 1 ± 0.4 | 1 ± 0.5 | 0.53 |
| Total cholesterol (mmol/L), mean ± SD |  |  |  |  | 4.4 ± 0.9 | 4.4 ± 0.9 | 4.4 ± 0.8 | 0.65 |
| LDL cholesterol (mmol/L), mean ± SD |  |  |  |  | 2.5 ± 0.7 | 2.4 ± 0.7 | 2.5 ± 0.7 | 0.49 |
| HDL cholesterol (mmol/L), mean ± SD |  |  |  |  | 1.5 ± 0.3 | 1.5 ± 0.3 | 1.5 ± 0.3 | 0.56 |
| Blood sugar (mmol/L), mean ± SD |  |  |  |  | 4.9 ± 0.5 | 4.9 ± 0.5 | 4.9 ± 0.4 | 0.68 |
| Insulin (pmol/L), mean ± SD |  |  |  |  | 43.5 ± 21.6 | 43 ± 23.4 | 44.5 ± 18.1 | 0.49 |
| Leptin (μg/L), mean ± SD |  |  |  |  | 11.8 ± 12.4 | 11.3 ± 11.9 | 12.7 ± 13.3 | 0.31 |
| Adiponectin (mg/L), mean ± SD |  |  |  |  | 8.9 ± 3.7 | 8.7 ± 3.5 | 9.4 ± 3.9 | 0.077 |
| HOMA IR index, mean ± SD |  |  |  |  | 1.4 ± 0.8 | 1.4 ± 0.8 | 1.4 ± 0.6 | 0.59 |
| HOMA β Index, mean ± SD |  |  |  |  | 95.4 ± 62.7 | 92.4 ± 58.9 | 100.8 ± 68.8 | 0.22 |
| Metabolic syndrome, n (%) |  |  |  |  | 140 (35) | 82 (32.2) | 58 (40) | 0.043 |

Abbreviations: BMI: Body mass index; DKK: Danish Krone. HbAA: Hemoglobin adducts of acrylamide; HbGA: Hemoglobin adducts glycidamide; HbEO: Hemoglobin adducts ethylene oxide; HOMA: homeostasis model assessment with HOMA IR: insulin resistance, HOMA β: pancreas beta cells activity, LDL: low density lipo-proteins; HDL: high density lipo-proteins.

Anthropometric measurements are based on clinical measurement at 20 years of age if available for the offspring or self-reported information when missing.

Large waist circumference indicates a waist circumference above 102 cm and 88 cm, respectively, for male and female offspring.

Overweight or obese indicate a BMI >25 kg/m^2^.

Metabolic syndrome was defined as having at least one ATP III criteria (waist circumference > 102 cm for males and > 88 cm for females; HDL cholesterol < 1.04 mmol/L for males and < 1.30 mmol/L for females; triglycerides ≥ 1.7 mmol/L, systolic blood pressure ≥ 130 mmHg or diastolic blood pressure ≥ 85 mmHg, or fasting blood sugar ≥ 6.1 mmol/L).

# Table S*3*: Study population characteristics by quartiles of hemoglobin adducts of acrylamide.

|  | Anthropometric outcomes (N=638) | | | | Metabolic biomarkers (N=400) | | | |
| --- | --- | --- | --- | --- | --- | --- | --- | --- |
|  | Lowest 1^st^ quartile  (n=186) | 2^nd^ quartile (n=168) | 3^rd^ quartile (n=145) | Highest 4^th^ quartile (n=139) | Lowest 1^st^ quartile (n=123) | 2^nd^ quartile (n=103) | 3^rd^ quartile (n=89) | Highest 4^th^ quartile (n=85) |
| Maternal characteristics |  |  |  |  |  |  |  |  |
| Maternal age (years), mean ± SD | 30.1 ± 4 | 29.1 ± 4.2 | 28.5 ± 3.6 | 28.8 ± 4.3 | 30.3 ± 4.2 | 29 ± 4.1 | 28.8 ± 3.7 | 28.4 ± 4 |
| Length of education, n (%) |  |  |  |  |  |  |  |  |
| Other or no education | 13 (7) | 24 (14.3) | 8 (5.5) | 15 (10.8) | 7 (5.7) | 17 (16.5) | 6 (6.7) | 11 (12.9) |
| Short education | 47 (25.3) | 43 (25.6) | 55 (37.9) | 66 (47.5) | 29 (23.6) | 21 (20.4) | 28 (31.5) | 43 (50.6) |
| Intermediate education | 88 (47.3) | 71 (42.3) | 56 (38.6) | 45 (32.4) | 61 (49.6) | 44 (42.7) | 34 (38.2) | 28 (32.9) |
| Academic education | 38 (20.4) | 30 (17.9) | 26 (17.9) | 13 (9.4) | 26 (21.1) | 21 (20.4) | 21 (23.6) | 3 (3.5) |
| Self-reported household income, n (%) |  |  |  |  |  |  |  |  |
| <150,000 | 30 (16.1) | 29 (17.3) | 26 (17.9) | 19 (13.7) | 21 (17.1) | 22 (21.4) | 13 (14.6) | 10 (11.8) |
| 150,000 to 200,000 | 32 (17.2) | 31 (18.5) | 23 (15.9) | 29 (20.9) | 20 (16.3) | 17 (16.5) | 15 (16.9) | 15 (17.6) |
| 200,000 to 300,000 | 56 (30.1) | 54 (32.1) | 50 (34.5) | 52 (37.4) | 37 (30.1) | 31 (30.1) | 30 (33.7) | 37 (43.5) |
| > 300,000 | 68 (36.6) | 54 (32.1) | 46 (31.7) | 39 (28.1) | 45 (36.6) | 33 (32) | 31 (34.8) | 23 (27.1) |
| Smoking pregnancy, n (%) | 24 (12.9) | 30 (17.9) | 66 (45.5) | 105 (75.5) | 15 (12.2) | 21 (20.4) | 45 (50.6) | 64 (75.3) |
| Healthy eating index, mean ± SD | 36.6 ± 6.4 | 34.5 ± 6.8 | 34.3 ± 7 | 32.5 ± 6.6 | 36 ± 6.4 | 35.1 ± 6.8 | 35.1 ± 6.9 | 31.6 ± 6.5 |
| HbAA (pmol/g Hb), mean ± SD | 52.9 ± 9.1 | 77.8 ± 6.9 | 115.8 ± 15.9 | 213.3 ± 70.7 | 53.1 ± 9 | 77.7 ± 6.9 | 117.4 ± 15.9 | 203.4 ± 49.7 |
| HbGA (pmol/g Hb), mean ± SD | 69 ± 24.3 | 93.9 ± 33.8 | 145 ± 73.1 | 254.8 ± 113.5 | 64.6 ± 22.3 | 92.4 ± 32.6 | 147.4 ± 76.7 | 246.2 ± 113.8 |
| HbEO (pmol/g Hb), mean ± SD | 69.5 ± 29 | 80.4 ± 38.4 | 140.2 ± 113.7 | 288.3 ± 195 | 68.8 ± 29.4 | 79.5 ± 35.2 | 145.9 ± 118.4 | 281.6 ± 195.9 |
| Pre-pregnancy BMI (kg/m^2^), mean ± SD | 21.6 ± 3 | 21.4 ± 2.6 | 21.4 ± 3.5 | 20.8 ± 2.1 | 21.8 ± 3.2 | 21.2 ± 2.5 | 21.6 ± 4 | 20.9 ± 2.1 |
| Overweight or Obese, n (%) | 20 (10.8) | 11 (6.5) | 12 (8.3) | 7 (5.0) | 13 (10.6) | 6 (5.8) | 8 (9.0) | 5 (5.9) |
| Offspring’s characteristics |  |  |  |  |  |  |  |  |
| Males, n (%) | 101 (54.3) | 70 (41.7) | 73 (50.3) | 65 (46.8) | 60 (48.8) | 34 (33) | 38 (42.7) | 30 (35.3) |
| Females, n (%) | 85 (45.7) | 98 (58.3) | 72 (49.7) | 74 (53.2) | 63 (51.2) | 69 (67) | 51 (57.3) | 55 (64.7) |
| Weight (kg), mean ± SD | 70.6 ± 11.5 | 68.3 ± 12.8 | 69.3 ± 12.1 | 70.8 ± 13.4 | 69.5 ± 11.7 | 66.2 ± 12.5 | 68 ± 12.6 | 69.8 ± 13.3 |
| Height (cm), mean ± SD | 177.2 ± 8.7 | 174.8 ± 10 | 175.6 ± 9.7 | 175.5 ± 9.8 | 175.9 ± 8.6 | 172.7 ± 8.7 | 174.2 ± 10 | 173.8 ± 9.4 |
| Waist circumference (cm), mean ± SD | 81.2 ± 8.6 | 81.6 ± 9.2 | 81.2 ± 8.2 | 84.6 ± 11.8 | 80.9 ± 8 | 80.1 ± 8.2 | 80.4 ± 7.8 | 84.1 ± 10.3 |
| Large waist circumference, n (%) | 13 (7) | 15 (8.9) | 11 (7.6) | 23 (16.5) | 8 (6.5) | 8 (7.8) | 5 (5.6) | 16 (18.8) |
| Body mass index (kg/m^2^), mean ± SD | 22.4 ± 2.8 | 22.2 ± 3 | 22.4 ± 2.9 | 22.9 ± 3.5 | 22.4 ± 2.8 | 22.1 ± 3 | 22.3 ± 2.9 | 23.1 ± 3.8 |
| Overweight or Obese, n (%) | 34 (18.3) | 27 (16.1) | 24 (16.6) | 31 (22.3) | 22 (17.9) | 14 (13.6) | 14 (15.7) | 23 (27.1) |
| Systolic blood pressure (mmHg), mean ± SD |  |  |  |  | 111.5 ± 10.2 | 108.8 ± 11.5 | 111.2 ± 10.7 | 111.4 ± 11.2 |
| Diastolic blood pressure (mmHg), mean ± SD |  |  |  |  | 66 ± 6.1 | 65.2 ± 6 | 65.2 ± 7.2 | 67.4 ± 7 |
| Triglycerides (mmol/L), mean ± SD |  |  |  |  | 1 ± 0.4 | 1 ± 0.4 | 1 ± 0.5 | 0.9 ± 0.4 |
| Total cholesterol (mmol/L), mean ± SD |  |  |  |  | 4.3 ± 0.8 | 4.5 ± 0.9 | 4.3 ± 0.9 | 4.5 ± 0.8 |
| LDL cholesterol (mmol/L), mean ± SD |  |  |  |  | 2.3 ± 0.6 | 2.6 ± 0.7 | 2.3 ± 0.8 | 2.6 ± 0.6 |
| HDL cholesterol (mmol/L), mean ± SD |  |  |  |  | 1.5 ± 0.3 | 1.5 ± 0.3 | 1.5 ± 0.3 | 1.4 ± 0.3 |
| Blood sugar (mmol/L), mean ± SD |  |  |  |  | 5 ± 0.4 | 4.8 ± 0.4 | 4.9 ± 0.5 | 5 ± 0.6 |
| Insulin (pmol/L), mean ± SD |  |  |  |  | 40.6 ± 17.6 | 44 ± 21.4 | 40.4 ± 17.5 | 50.6 ± 28.8 |
| Leptin (μg/L), mean ± SD |  |  |  |  | 10.9 ± 12.4 | 11.9 ± 11.7 | 11.1 ± 12.7 | 13.7 ± 13 |
| Adiponectin (mg/L), mean ± SD |  |  |  |  | 8.5 ± 4 | 9.1 ± 3.1 | 9 ± 3.8 | 9.2 ± 3.7 |
| HOMA IR index, mean ± SD |  |  |  |  | 1.3 ± 0.6 | 1.4 ± 0.7 | 1.3 ± 0.6 | 1.6 ± 1.1 |
| HOMA β Index, mean ± SD |  |  |  |  | 85.6 ± 47.1 | 101.1 ± 66.5 | 93.8 ± 78.2 | 104.4 ± 58.3 |
| Metabolic syndrome, n (%) |  |  |  |  | 35 (28.5) | 36 (35) | 28 (31.5) | 41 (48.2) |

Abbreviations: BMI: Body mass index; DKK: Danish Krone. HbAA: Hemoglobin adducts of acrylamide; HbGA: Hemoglobin adducts glycidamide; HbEO: Hemoglobin adducts ethylene oxide; HOMA: homeostasis model assessment with HOMA IR: insulin resistance, HOMA β: pancreas beta cells activity, LDL: low density lipo-proteins; HDL: high density lipo-proteins. Anthropometric measurements are based on clinical measurement if available or self-reported information when missing. Large waist circumference indicates a waist circumference above 102 or 88 for male or female offspring. Metabolic syndrome was defined as having at least one ATP III criteria (waist circumference > 102 cm for males and > 88 cm for females; HDL cholesterol < 1.04 mmol/L for males and < 1.30 mmol/L for females; triglycerides ≥ 1.7 mmol/L, systolic blood pressure ≥ 130 mmHg or diastolic blood pressure ≥ 85 mmHg, or fasting blood sugar ≥ 6.1 mmol/L).

# Table S*4*: Study population characteristics by sex and metabolic health outcome.

|  | Anthropometric outcomes | | | | Metabolic biomarkers | | | |
| --- | --- | --- | --- | --- | --- | --- | --- | --- |
|  | Overall (n=638) | Male (n=309) | Female (n=329) | p-value | Overall (n=400) | Male (n=162) | Female (n=238) | p-value |
| Maternal characteristics |  |  |  |  |  |  |  |  |
| Maternal age (years), mean ± SD | 29.2 ± 4.1 | 29.4 ± 4.3 | 28.9 ± 3.9 | 0.17 | 29.2 ± 4.1 | 29.7 ± 4.3 | 28.9 ± 3.9 | 0.064 |
| Length of education, n (%) |  |  |  | 0.22 |  |  |  | 0.22 |
| Other or no education | 60 (9.4) | 31 (10) | 29 (8.8) |  | 41 (10.2) | 19 (11.7) | 22 (9.2) |  |
| Short education | 211 (33.1) | 95 (30.7) | 116 (35.3) |  | 121 (30.2) | 45 (27.8) | 76 (31.9) |  |
| Intermediate education | 260 (40.8) | 137 (44.3) | 123 (37.4) |  | 167 (41.8) | 75 (46.3) | 92 (38.7) |  |
| Academic education | 107 (16.8) | 46 (14.9) | 61 (18.5) |  | 71 (17.8) | 23 (14.2) | 48 (20.2) |  |
| Household income (DKK), n (%) |  |  |  | 0.80 |  |  |  | 0.53 |
| <150,000 | 104 (16.3) | 50 (16.2) | 54 (16.4) |  | 66 (16.5) | 32 (19.8) | 34 (14.3) |  |
| 150,000 to 200,000 | 115 (18) | 60 (19.4) | 55 (16.7) |  | 67 (16.8) | 27 (16.7) | 40 (16.8) |  |
| 200,000 to 300,000 | 212 (33.2) | 103 (33.3) | 109 (33.1) |  | 135 (33.8) | 51 (31.5) | 84 (35.3) |  |
| > 300,000 | 207 (32.4) | 96 (31.1) | 111 (33.7) |  | 132 (33) | 52 (32.1) | 80 (33.6) |  |
| Healthy eating index, mean ± SD | 34.6 ± 6.8 | 34.8 ± 6.9 | 34.5 ± 6.8 | 0.51 | 34.6 ± 6.8 | 35.1 ± 6.9 | 34.4 ± 6.8 | 0.28 |
| Pre-pregnancy body mass index (kg/m^2^), mean ± SD | 21.3 ± 2.9 | 21.4 ± 2.9 | 21.3 ± 2.8 | 0.83 | 21.4 ± 3 | 21.5 ± 3.4 | 21.4 ± 2.8 | 0.64 |
| Overweight or Obese, n (%) | 50 (7.8) | 24 (7.8) | 26 (7.9) | 0.78 | 32 (8) | 13 (8) | 19 (8) | 0.29 |
| Smoking pregnancy, n (%) | 225 (35.3) | 103 (33.3) | 122 (37.1) | 0.21 | 145 (36.2) | 54 (33.3) | 91 (38.2) | 0.0021 |
| Acrylamide Hb adducts (pmol/g), mean ± SD | 108.7 ± 68.8 | 105.7 ± 70.5 | 111.5 ± 67.2 | 0.29 | 105.7 ± 61.1 | 98 ± 55.3 | 110.9 ± 64.3 | 0.033 |
| Glycidamide Hb adducts (pmol/g), mean ± SD | 166.3 ± 111.3 | 160.8 ± 115.3 | 171.4 ± 107.7 | 0.46 | 164.9 ± 110.2 | 142.6 ± 94.7 | 180.2 ± 117.8 | 0.034 |
| Ethylene oxide Hb adducts (pmol/g), mean ± SD | 136.1 ± 137.7 | 135.2 ± 154.7 | 137 ± 119.8 | 0.87 | 133.9 ± 136 | 131.9 ± 150.6 | 135.3 ± 125.5 | 0.81 |
| Offspring’s characteristics |  |  |  |  |  |  |  |  |
| Males, n (%) | 309 (48.4) |  |  |  | 162 (40.5) |  |  |  |
| Females, n (%) | 329 (51.6) |  |  |  | 238 (59.5) |  |  |  |
| Weight (kg), mean ± SD | 69.8 ± 12.4 | 76.5 ± 10.8 | 63.4 ± 10.4 | <0.001 | 68.4 ± 12.5 | 76.2 ± 11.1 | 63.1 ± 10.4 | <0.001 |
| Height (cm), mean ± SD | 175.8 ± 9.5 | 183.2 ± 6.8 | 168.9 ± 5.7 | <0.001 | 174.2 ± 9.2 | 182.6 ± 6.5 | 168.5 ± 5.7 | <0.001 |
| Waist circumference (cm), mean ± SD | 82.1 ± 9.5 | 84.5 ± 9.3 | 79.8 ± 9.2 | <0.001 | 81.3 ± 8.6 | 83.5 ± 8 | 79.8 ± 8.8 | <0.001 |
| Large waist circumference, n (%) | 62 (9.7) | 11 (3.6) | 51 (15.5) | <0.001 | 37 (9.2) | 3 (1.9) | 34 (14.3) | <0.001 |
| Body mass index (kg/m^2^), mean ± SD | 22.5 ± 3.1 | 22.8 ± 2.9 | 22.2 ± 3.2 | 0.017 | 22.4 ± 3.1 | 22.8 ± 3 | 22.2 ± 3.2 | 0.0319 |
| Overweight or Obese, n (%) | 116 (18.2) | 57 (18.4) | 59 (17.9) | 0.85 | 73 (18.2) | 34 (21) | 39 (16.4) | 0.56 |
| Systolic blood pressure (mmHg), mean ± SD |  |  |  |  | 110.7 ± 10.9 | 118.5 ± 9.5 | 105.4 ± 8.3 | <0.001 |
| Diastolic blood pressure (mmHg), mean ± SD |  |  |  |  | 65.9 ± 6.6 | 65 ± 7 | 66.5 ± 6.2 | 0.021 |
| Triglycerides (mmol/l), mean ± SD |  |  |  |  | 1 ± 0.4 | 0.9 ± 0.4 | 1 ± 0.5 | <0.001 |
| Total cholesterol (mmol/L), mean ± SD |  |  |  |  | 4.4 ± 0.9 | 4 ± 0.7 | 4.6 ± 0.9 | <0.001 |
| LDL cholesterol (mmol/L), mean ± SD |  |  |  |  | 2.5 ± 0.7 | 2.3 ± 0.6 | 2.5 ± 0.8 | 0.0031 |
| HDL cholesterol (mmol/L), mean ± SD |  |  |  |  | 1.5 ± 0.3 | 1.3 ± 0.3 | 1.6 ± 0.3 | <0.001 |
| Blood sugar (mmol/L), mean ± SD |  |  |  |  | 4.9 ± 0.5 | 5.1 ± 0.4 | 4.8 ± 0.5 | <0.001 |
| Insulin (pmol/L), mean ± SD |  |  |  |  | 43.5 ± 21.6 | 41.2 ± 19.1 | 45.2 ± 23.1 | 0.061 |
| Leptin (μg/L), mean ± SD |  |  |  |  | 11.8 ± 12.4 | 3.9 ± 5.3 | 17.2 ± 13 | <0.001 |
| Adiponectin (mg/L), mean ± SD |  |  |  |  | 8.9 ± 3.7 | 7.4 ± 3.4 | 10 ± 3.5 | <0.001 |
| HOMA IR index, mean ± SD |  |  |  |  | 1.4 ± 0.8 | 1.4 ± 0.7 | 1.4 ± 0.8 | 0.60 |
| HOMA β Index, mean ± SD |  |  |  |  | 95.4 ± 62.7 | 76.7 ± 43.2 | 108.2 ± 70.3 | <0.001 |
| Metabolic syndrome, n (%) |  |  |  |  | 140 (35) | 46 (28.4) | 94 (39.5) | <0.001 |

Abbreviations: BMI: Body mass index; DKK: Danish Krone. HbAA: Hemoglobin adducts of acrylamide; HbGA: Hemoglobin adducts glycidamide; HbEO: Hemoglobin adducts ethylene oxide;

HOMA: homeostasis model assessment with HOMA IR: insulin resistance, HOMA β: pancreas beta cells activity, LDL: low density lipo-proteins; HDL: high density lipo-proteins.

Anthropometric measurements are based on clinical measurement if available or self-reported information when missing. Large waist circumference indicates a waist circumference above 102 or 88 for male or female offspring. Metabolic syndrome was defined as having at least one ATP III criteria (waist circumference > 102 cm for males and > 88 cm for females; HDL cholesterol < 1.04 mmol/L for males and < 1.30 mmol/L for females; triglycerides ≥ 1.7 mmol/L, systolic blood pressure ≥ 130 mmHg or diastolic blood pressure ≥ 85 mmHg, or fasting blood sugar ≥ 6.1 mmol/L).

# Table S*5*: Associations between prenatal exposure to acrylamide and metabolic status at 20 years of age with increasing degree of adjustments.

| Outcome | n | Model 1 | Model 2 | Model 3 (final) | Model 4 |
| --- | --- | --- | --- | --- | --- |
|  |  | β (95% CI) | β (95% CI) | β (95% CI) |  |
| Weight | 638 | −0.06 (−0.2, 0.08) | −0.09 (−0.2, 0.08) | −0.08 (−0.2, 0.08) | −0.07 (−0.2, 0.1) |
| Height | 638 | −0.1 (−0.2, 0.009) | −0.07 (−0.2, 0.06) | −0.07 (−0.2, 0.05) | −0.06 (−0.2, 0.07) |
| Waist circumference | 638 | 0.1 (−0.01, 0.2) | 0.06 (−0.06, 0.2) | 0.06 (−0.07, 0.2) | 0.09 (−0.04, 0.2) |
| Waist circumference above 102 or 88^1^ | 638 | 1.02 (0.99, 1.06) | 1.00 (0.96, 1.04) | 1.00 (0.96, 1.04) | 1.01 (0.96, 1.05) |
| Body mass index | 638 | 0.004 (−0.03, 0.04) | −0.01 (−0.05, 0.03) | −0.01 (−0.05, 0.03) | −0.007 (−0.05, 0.03) |
| Overweight or Obese^1^ | 638 | 1.00 (0.97, 1.03) | 0.98 (0.95, 1.02) | 0.98 (0.95, 1.02) | 0.99 (0.95, 1.02) |
| Systolic blood pressure | 400 | −0.04 (−0.2, 0.1) | −0.1 (−0.3, 0.08) | −0.1 (−0.3, 0.09) | −0.1 (−0.3, 0.1) |
| Diastolic blood pressure | 400 | 0.03 (−0.08, 0.1) | −0.02 (−0.1, 0.1) | −0.02 (−0.2, 0.1) | 0.003 (−0.1, 0.1) |
| Triglycerides | 400 | −0.0006 (−0.008, 0.007) | −0.002 (−0.01, 0.006) | −0.002 (−0.01, 0.007) | −0.002 (−0.01, 0.007) |
| Total cholesterol | 400 | 0.008 (−0.006, 0.02) | 0.01 (−0.007, 0.03) | 0.008 (−0.009, 0.02) | 0.008 (−0.009, 0.03) |
| LDL cholesterol | 400 | 0.01 (−0.0005, 0.02) | 0.01 (−0.00003, 0.03) | 0.01 (−0.002, 0.03) | 0.01 (−0.002, 0.03) |
| HDL cholesterol | 400 | −0.003 (−0.008, 0.003) | −0.003 (−0.009, 0.004) | −0.003 (−0.01, 0.003) | −0.003 (−0.01, 0.003) |
| Blood sugar | 400 | 0.002 (−0.006, 0.01) | 0.003 (−0.007, 0.01) | 0.004 (−0.005, 0.01) | 0.004 (−0.005, 0.01) |
| Insulin^2^ | 400 | 0.35 (−0.38, 1.10) | 0.40 (−0.46, 1.26) | 0.36 (−0.51, 1.24) | 0.38 (−0.49, 1.26) |
| Leptin^2^ | 400 | 1.13 (−0.90, 3.20) | 1.23 (−1.14, 3.65) | 1.10 (−1.30, 3.55) | 1.10 (−1.30, 3.57) |
| Adiponectin^2^ | 400 | 0.44 (−0.24, 1.13) | 0.21 (−0.58, 1.01) | 0.11 (−0.70, 0.92) | 0.11 (−0.70, 0.92) |
| HOMA IR index^2^ | 400 | 0.39 (−0.39, 1.17) | 0.44 (−0.47, 1.36) | 0.43 (−0.49, 1.36) | 0.45 (−0.48, 1.38) |
| HOMA β Index^2^ | 400 | 0.79 (−0.23, 1.83) | 0.68 (−0.51, 1.89) | 0.58 (−0.62, 1.80) | 0.48 (−0.72, 1.70) |
| Metabolic syndrome (one criteria or more)^1^ | 400 | 1.04 (1.00, 1.07) | 1.03 (0.99, 1.08) | 1.04 (0.99, 1.08) | 1.04 (1.00, 1.08) |

Abbreviations: HOMA: homeostasis model assessment with HOMA IR: insulin resistance, HOMA β: pancreas beta cells activity, LDL: low density lipo-proteins; HDL: high density lipo-proteins.

^1^ OR (95% CI). ^2^ Natural logarithm transformed outcome; estimates expressed as percentage of change (95% CI). Linear estimates are expressed by 10 pmol/g increase in hemoglobin adducts of acrylamide (HbAA).

Model 1: adjusted for household income, maternal education and maternal age in pregnancy.

Model 2: Model 1 further adjusted for maternal smoking in pregnancy.

Model 3 (final model): Model 2 further adjusted for maternal healthy eating index in pregnancy.

Model 4: Model 3 further adjusted for maternal overweight or obesity

# Table S6: Association between prenatal exposure to glycidamide and metabolic status at 20 years of age with increasing degree of adjustments.

| Outcome | n | Model 1 | Model 2 | Model 3 (main model) | ‍Model 4 |
| --- | --- | --- | --- | --- | --- |
|  |  | β (95% CI) | β (95% CI) | β (95% CI) | ‍β (95% CI) |
| Weight | 241 | −0.08 (−0.2, 0.07) | −0.08 (−0.2, 0.09) | −0.07 (−0.2, 0.1) | −0.07 (−0.2, 0.1) |
| Height | 241 | −0.07 (−0.2, 0.04) | −0.04 (−0.2, 0.09) | −0.04 (−0.2, 0.08) | −0.04 (−0.2, 0.08) |
| Waist circumference | 241 | 0.03 (−0.1, 0.2) | 0.008 (−0.1, 0.1) | 0.008 (−0.1, 0.1) | 0.008 (−0.1, 0.1) |
| Waist circumference above 102 or 88*^1^* | 241 | 1 (1, 1) | 1 (1, 1) | 1 (1, 1) | 1.00 (0.96, 1.03) |
| Body mass index | 241 | −0.01 (−0.05, 0.03) | −0.02 (−0.06, 0.02) | −0.02 (−0.06, 0.03) | −0.02 (−0.06, 0.03) |
| Overweight or Obese*^1^* | 241 | 1 (1, 1) | 1 (0.9, 1) | 1 (0.9, 1) | 0.97 (0.94, 1.01) |
| Systolic blood pressure | 148 | −0.1 (−0.3, 0.02) | −0.2 (−0.4, 0.01) | −0.2 (−0.4, 0.01) | −0.2 (−0.4, 0.01) |
| Diastolic blood pressure | 148 | −0.03 (−0.1, 0.08) | −0.05 (−0.2, 0.07) | −0.06 (−0.2, 0.07) | −0.06 (−0.2, 0.07) |
| Triglycerides | 148 | 0.0008 (−0.006, 0.008) | −0.0006 (−0.008, 0.007) | 0.001 (−0.005, 0.008) | −0.00002 (−0.008, 0.008) |
| Total cholesterol | 148 | 0.01 (0.002, 0.03) | 0.02 (0.001, 0.03) | 0.01 (−0.0001, 0.03) | 0.01 (−0.0001, 0.03) |
| LDL cholesterol | 148 | 0.01 (0.005, 0.02) | 0.02 (0.005, 0.03) | 0.02 (0.004, 0.03) | 0.02 (0.004, 0.03) |
| HDL cholesterol | 148 | −0.0008 (−0.006, 0.004) | −0.0007 (−0.006, 0.005) | −0.001 (−0.007, 0.005) | −0.001 (−0.007, 0.005) |
| Blood sugar | 148 | −0.004 (−0.01, 0.002) | −0.005 (−0.01, 0.002) | −0.005 (−0.01, 0.002) | −0.005 (−0.01, 0.002) |
| Insulin | 148 | −0.09 (−0.8, 0.6) | 0.03 (−0.7, 0.8) | −0.0003 (−0.8, 0.8) | 0.00 (−0.76, 0.77) |
| Leptin*^2^* | 148 | 0.7 (−1, 3) | 0.5 (−2, 3) | 0.6 (−2, 3) | 0.55 (−1.52, 2.66) |
| Adiponectin*^2^* | 148 | 0.4 (−0.3, 1) | 0.4 (−0.3, 1) | 0.3 (−0.4, 1) | 0.29 (−0.42, 1.00) |
| HOMA IR index^2^ | 148 | −0.2 (−0.9, 0.5) | −0.08 (−0.9, 0.7) | −0.1 (−0.9, 0.7) | −0.11 (−0.91, 0.69) |
| HOMA β Index^2^ | 148 | 0.2 (−0.5, 1) | 0.4 (−0.4, 1) | 0.5 (−0.4, 1) | 0.45 (−0.41, 1.32) |
| Metabolic syndrome (one criteria or more)*^1^* | 148 | 1 (1, 1) | 1 (1, 1) | 1 (1, 1) | 1.01 (0.98, 1.05) |

Abbreviations: HOMA: homeostasis model assessment with HOMA IR: insulin resistance, HOMA β: pancreas beta cells activity, LDL: low density lipo-proteins; HDL: high density lipo-proteins.

^1^ OR (95% CI). ^2^ Natural logarithm transformed outcome; estimates expressed as percentage of change (95% CI). Linear estimates are expressed by 10 pmol/g increase in hemoglobin adducts of glycidamide (HbGA).

Model 1: adjusted for household income, maternal education and maternal age in pregnancy.

Model 2: Model 1 further adjusted for maternal smoking in pregnancy.

Model 3 (final model): Model 2 further adjusted for maternal healthy eating index in pregnancy.
Model 4: Model 3 further adjusted for maternal overweight or obesity.

# Table S7: Associations between prenatal exposure to acrylamide as quartile and metabolic health at 20 years of age.

| Outcome |  | 1^st^ quartile (lowest) | 2^nd^ quartile | 3^rd^ quartile | 4^th^ quartile (highest) of HbAA |
| --- | --- | --- | --- | --- | --- |
| Continuous scale outcomes | N | β (95% CI) | β (95% CI) | β (95% CI) | β (95% CI) |
| Weight | 638 | Ref | −2 (−5, 0.3) | −2 (−4, 1) | −0.2 (−3, 3) |
| Height | 638 | Ref | −2 (−4, −0.4) | −0.8 (−3, 1) | −0.8 (−3, 2) |
| Waist circumference | 638 | Ref | 0.1 (−2, 2) | −0.8 (−3, 1) | 2 (−0.4, 4) |
| Body mass index | 638 | Ref | −0.2 (−0.8, 0.5) | −0.3 (−1, 0.4) | 0.1 (−0.6, 0.9) |
| Systolic blood pressure | 400 | Ref | −3 (−6, 0.09) | −0.5 (−4, 3) | −0.8 (−4, 3) |
| Diastolic blood pressure | 400 | Ref | −0.8 (−3, 0.9) | −1 (−3, 0.5) | 0.2 (−2, 2) |
| Triglycerides | 400 | Ref | −0.02 (−0.1, 0.1) | 0.008 (−0.1, 0.1) | −0.07 (−0.2, 0.07) |
| Total cholesterol | 400 | Ref | 0.3 (0.04, 0.5) | −0.03 (−0.3, 0.2) | 0.1 (−0.2, 0.4) |
| LDL cholesterol | 400 | Ref | 0.3 (0.07, 0.4) | −0.008 (−0.2, 0.2) | 0.2 (0.01, 0.5) |
| HDL cholesterol | 400 | Ref | 0.02 (−0.07, 0.1) | −0.03 (−0.1, 0.06) | −0.07 (−0.2, 0.03) |
| Blood sugar | 400 | Ref | −0.1 (−0.2, 0.004) | 0.002 (−0.1, 0.1) | 0.08 (−0.07, 0.2) |
| Insulin* | 400 | Ref | 5.99 (−5.59, 19.00) | −1.33 (−13.04, 11.95) | 15.02 (−0.23, 32.59) |
| Leptin* | 400 | Ref | 40.11 (1.80, 92.83) | 0.51 (−29.06, 42.42) | 31.21 (−11.37, 94.26) |
| Adiponectin* | 400 | Ref | 9.09 (−2.05, 21.51) | 5.01 (−6.64, 18.11) | 3.71 (−9.15, 18.40) |
| HOMA IR index* | 400 | Ref | 3.22 (−8.72, 16.72) | −1.45 (−13.82, 12.70) | 16.50 (0.17, 35.49) |
| HOMA β Index* | 400 | Ref | 7.75 (−8.31, 26.64) | 1.91 (−14.55, 21.55) | 13.46 (−6.96, 38.36) |
|  |  |  |  |  |  |
| Categorical outcomes |  | OR (95% CI) | OR (95% CI) | OR (95% CI) | OR (95% CI) |
| Overweight or Obese | 638 | Ref | 0.86 (0.48, 1.51) | 0.71 (0.38, 1.31) | 0.93 (0.48, 1.78) |
| Large waist | 638 | Ref | 1.23 (0.55, 2.74) | 0.74 (0.30, 1.80) | 1.44 (0.62, 3.42) |
| Metabolic syndrome | 400 | Ref | 0.86 (0.48, 1.51) | 0.71 (0.38, 1.31) | 0.93 (0.48, 1.78) |

Abbreviations: HOMA: homeostasis model assessment with HOMA IR: insulin resistance, HOMA β: pancreas beta cells activity, LDL: low density lipo-proteins; HDL: high density lipo-proteins. HbAA: Hemoglobin adducts of acrylamide.

Average levels of each outcome or number of cases can be found in Table S4.

Overweight or Obese: BMI ≥ 25 kg/m^2^. Large waist: Waist circumference above 102 or 88 cm for male or female. Metabolic syndrome was defined as having at least one ATP III criteria (waist circumference > 102 cm for males and > 88 cm for females; HDL cholesterol < 1.04 mmol/L for males and < 1.30 mmol/L for females; triglycerides ≥ 1.7 mmol/L, systolic blood pressure ≥ 130 mmHg or diastolic blood pressure ≥ 85 mmHg, or fasting blood sugar ≥ 6.1 mmol/L).

Estimates are from models adjusted for household income, maternal education, maternal age, maternal smoking, and maternal healthy eating index in pregnancy.

* Natural logarithm transformed outcome; estimates expressed as percentage of change (95% CI)

# Table S*8*: Associations between acrylamide and glycidamide hemoglobin adducts and sex-specific Z-scores of metabolic outcomes with increasing levels of adjustment.

|  | n | Model 1 | Model 2 | Model 3 |
| --- | --- | --- | --- | --- |
|  |  | β (95% CI) | β (95% CI) | β (95% CI) |
| HbAA(pmol/g Hb) |  |  |  |  |
| Weight | 638 | −0.003 (−0.01, 0.009) | −0.006 (−0.02, 0.007) | −0.006 (−0.02, 0.007) |
| Height | 638 | −0.01 (−0.02, 0.001) | −0.006 (−0.02, 0.007) | −0.007 (−0.02, 0.006) |
| Waist circumference | 638 | 0.01 (0.0006, 0.02) | 0.008 (−0.005, 0.02) | 0.007 (−0.006, 0.02) |
| Body mass index | 638 | 0.002 (−0.009, 0.01) | −0.003 (−0.02, 0.01) | −0.002 (−0.02, 0.01) |
| Systolic blood pressure | 400 | 0.006 (−0.01, 0.02) | −0.003 (−0.02, 0.02) | −0.003 (−0.02, 0.02) |
| Diastolic blood pressure | 400 | 0.002 (−0.01, 0.02) | −0.004 (−0.02, 0.02) | −0.005 (−0.02, 0.01) |
| Blood sugar | 400 | 0.01 (−0.007, 0.03) | 0.01 (−0.008, 0.03) | 0.02 (−0.005, 0.04) |
| Triglycerides | 400 | −0.004 (−0.02, 0.01) | −0.009 (−0.03, 0.01) | −0.007 (−0.03, 0.01) |
| Total cholesterol | 400 | 0.007 (−0.01, 0.02) | 0.009 (−0.01, 0.03) | 0.007 (−0.01, 0.03) |
| LDL cholesterol | 400 | 0.02 (−0.001, 0.03) | 0.02 (−0.0008, 0.04) | 0.02 (−0.003, 0.04) |
| HDL cholesterol | 400 | −0.02 (−0.03, 0.001) | −0.02 (−0.03, 0.004) | −0.02 (−0.04, 0.004) |
| Adiponectin | 400 | 0.005 (−0.01, 0.02) | −0.002 (−0.02, 0.02) | −0.004 (−0.02, 0.02) |
| Insulin | 400 | 0.006 (−0.01, 0.02) | 0.01 (−0.009, 0.03) | 0.01 (−0.009, 0.03) |
| Leptin | 400 | −0.004 (−0.02, 0.01) | −0.002 (−0.02, 0.02) | −0.005 (−0.02, 0.02) |
|  |  |  |  |  |
| HbGA (pmol/g Hb) |  |  |  |  |
| Weight | 241 | −0.005 (−0.02, 0.007) | −0.006 (−0.02, 0.007) | −0.006 (−0.02, 0.008) |
| Height | 241 | −0.006 (−0.02, 0.006) | −0.003 (−0.02, 0.01) | −0.003 (−0.02, 0.01) |
| Waist circumference | 241 | 0.004 (−0.009, 0.02) | 0.001 (−0.01, 0.02) | 0.001 (−0.01, 0.02) |
| Body mass index | 241 | −0.002 (−0.01, 0.01) | −0.005 (−0.02, 0.008) | −0.005 (−0.02, 0.009) |
| Systolic blood pressure | 148 | −0.006 (−0.02, 0.01) | −0.01 (−0.03, 0.009) | −0.01 (−0.03, 0.008) |
| Diastolic blood pressure | 148 | −0.006 (−0.02, 0.01) | −0.01 (−0.03, 0.009) | −0.01 (−0.03, 0.008) |
| Blood sugar | 148 | −0.003 (−0.02, 0.01) | −0.006 (−0.02, 0.01) | −0.007 (−0.02, 0.009) |
| Triglycerides | 148 | −0.002 (−0.02, 0.01) | −0.005 (−0.02, 0.01) | −0.003 (−0.02, 0.02) |
| Total cholesterol | 148 | 0.01 (−0.003, 0.03) | 0.01 (−0.003, 0.03) | 0.01 (−0.005, 0.03) |
| LDL cholesterol | 148 | 0.02 (0.004, 0.03) | 0.02 (0.005, 0.04) | 0.02 (0.004, 0.04) |
| HDL cholesterol | 148 | −0.01 (−0.02, 0.005) | −0.008 (−0.02, 0.009) | −0.009 (−0.03, 0.008) |
| Adiponectin | 148 | 0.007 (−0.009, 0.02) | 0.007 (−0.01, 0.02) | 0.006 (−0.01, 0.02) |
| Insulin | 148 | −0.004 (−0.02, 0.01) | 0.00002 (−0.02, 0.02) | −0.0003 (−0.02, 0.02) |
| Leptin | 148 | −0.003 (−0.02, 0.02) | −0.0003 (−0.02, 0.02) | −0.0007 (−0.02, 0.02) |

Abbreviations: HbAA: Hemoglobin adducts of acrylamide, HbGA: Hemoglobin adducts of glycidamide. HOMA: homeostasis model assessment with HOMA IR: insulin resistance, HOMA β: pancreas beta cells activity, LDL: low density lipo-proteins; HDL: high density lipo-proteins.

^1^ OR (95% CI). ^2^ Natural logarithm transformed outcome; estimates expressed as percentage of change (95% CI). Linear estimates are expressed by 10 pmol/g increase in hemoglobin adducts of acrylamide (HbAA) or glycidamide (HbGA).

Model 1: adjusted for household income, maternal education and maternal age in pregnancy.

Model 2: Model 1 further adjusted for maternal smoking in pregnancy.

Model 3 (final model): Model 2 further adjusted for maternal healthy eating index in pregnancy.

# Table S9. Association between prenatal exposure to HbAA and anthropometrics outcomes for the whole population (Base models) and among offspring with clinical measurements only.

|  | Self-measured and clinical measurement data (n = 638) | | Clinical measurements data (n = 401) | | |
| --- | --- | --- | --- | --- | --- |
| Outcome | Model 1 | Model 3 (final) | Model 1 | Model 3 (final) | |
|  | β (95% CI) | β (95% CI) | β (95% CI) | β (95% CI) | |
| Weight | −0.06 (−0.2, 0.08) | −0.08 (−0.2, 0.08) | −0.09 (−0.3, 0.1) | −0.1 (−0.4, 0.1) | |
| Height | −0.1 (−0.2, 0.009) | −0.07 (−0.2, 0.05) | −0.1 (−0.3, 0.01) | −0.1 (−0.3, 0.05) | |
| Waist circumference | 0.1 (−0.01, 0.2) | 0.06 (−0.07, 0.2) | 0.08 (−0.06, 0.2) | 0.07 (−0.1, 0.2) | |
| Waist circumference above 102 or 88^1^ | 1.02 (0.99, 1.06) | 1.00 (0.96, 1.04) | 1.04 (0.98, 1.09) | 1.03 (0.96, 1.09) | |
| Body mass index | 0.004 (−0.03, 0.04) | −0.01 (−0.05, 0.03) | 0.005 (−0.05, 0.06) | −0.01 (−0.07, 0.05) | |
| Overweight or Obese^1^ | 1.00 (0.97, 1.03) | 0.98 (0.95, 1.02) | 1.02 (0.98, 1.06) | 1.00 (0.95, 1.05) | |
| ^1^ OR (95% CI)  Estimates are from models adjusted for household income, maternal education, maternal age, maternal smoking, and maternal healthy eating index in pregnancy. | | | | |  |

# Table S10. Associations between prenatal exposure to acrylamide and metabolic health outcomes – comparing results of models with single adducts and the sum of acrylamide and glycidamide hemoglobin adducts.

|  | HbAA | | HbGA | | | Sum HbAA + HbGA | | | | |
| --- | --- | --- | --- | --- | --- | --- | --- | --- | --- | --- |
| Outcome | Model 1 (N=638) | Model 3 (N=400) | Model 1 (N=241) | Model 3 (N=148) | | Model 1 (N=241) | Model 3 (N=148) | | | |
|  | β (95% CI) | β (95% CI) | β (95% CI) | β (95% CI) | | β (95% CI) | β (95% CI) | | | |
| Weight | −0.06 (−0.2, 0.08) | −0.08 (−0.2, 0.08) | −0.08 (−0.2, 0.07) | −0.07 (−0.2, 0.1) | | −0.06 (−0.1, 0.03) | −0.04 (−0.1, 0.06) | | | |
| Height | −0.1 (−0.2, 0.009) | −0.07 (−0.2, 0.05) | −0.07 (−0.2, 0.04) | −0.04 (−0.2, 0.08) | | −0.04 (−0.1, 0.03) | −0.02 (−0.1, 0.06) | | | |
| Waist circumference | 0.1 (−0.01, 0.2) | 0.06 (−0.07, 0.2) | 0.03 (−0.1, 0.2) | 0.008 (−0.1, 0.1) | | 0.03 (−0.05, 0.1) | 0.02 (−0.08, 0.1) | | | |
| Waist circumference above 102 or 88*1* | 1.02 (0.99, 1.06) | 1.00 (0.96, 1.04) | 1.01 (0.97, 1.04) | 1.00 (0.96, 1.03) | | 1.00 (0.98, 1.02) | 1.00 (0.97, 1.02) | | | |
| Body mass index | 0.004 (−0.03, 0.04) | −0.01 (−0.05, 0.03) | −0.01 (−0.05, 0.03) | −0.02 (−0.06, 0.03) | | −0.009 (−0.03, 0.02) | −0.009 (−0.04, 0.02) | | | |
| Overweight or Obese*1* | 1.00 (0.97, 1.03) | 0.98 (0.95, 1.02) | 0.98 (0.95, 1.01) | 0.97 (0.94, 1.01) | | 0.99 (0.97, 1.01) | 0.98 (0.96, 1.01) | | | |
| Systolic blood pressure | −0.04 (−0.2, 0.1) | −0.1 (−0.3, 0.09) | −0.1 (−0.3, 0.02) | −0.2 (−0.4, 0.01) | | −0.07 (−0.2, 0.03) | −0.09 (−0.2, 0.04) | | | |
| Diastolic blood pressure | 0.03 (−0.08, 0.1) | −0.02 (−0.2, 0.1) | −0.03 (−0.1, 0.08) | −0.06 (−0.2, 0.07) | | −0.0008 (−0.07, 0.07) | −0.02 (−0.1, 0.06) | | | |
| Triglycerides | −0.0006 (−0.008, 0.007) | −0.002 (−0.01, 0.007) | 0.0008 (−0.006, 0.008) | −0.00002 (−0.008, 0.008) | | 0.001 (−0.003, 0.005) | 0.0005 (−0.005, 0.006) | | | |
| Total cholesterol | 0.008 (−0.006, 0.02) | 0.008 (−0.009, 0.02) | 0.01 (0.002, 0.03) | 0.01 (−0.0001, 0.03) | | 0.009 (0.001, 0.02) | 0.01 (0.0003, 0.02) | | | |
| LDL cholesterol | 0.01 (−0.0005, 0.02) | 0.01 (−0.002, 0.03) | 0.01 (0.005, 0.02) | 0.02 (0.004, 0.03) | | 0.009 (0.002, 0.02) | 0.009 (0.002, 0.02) | | | |
| HDL cholesterol | −0.003 (−0.008, 0.003) | −0.003 (−0.01, 0.003) | −0.0008 (−0.006, 0.004) | −0.001 (−0.007, 0.005) | | 0.0002 (−0.003, 0.003) | 0.0003 (−0.003, 0.004) | | | |
| Blood sugar measured with Accu-Chek | 0.002 (−0.006, 0.01) | 0.004 (−0.005, 0.01) | −0.004 (−0.01, 0.002) | −0.005 (−0.01, 0.002) | | −0.002 (−0.006, 0.002) | −0.003 (−0.007, 0.002) | | | |
| Insulin*2* | 0.35 (−0.38, 1.10) | 0.36 (−0.51, 1.24) | −0.09 (−0.75, 0.58) | 0.00 (−0.76, 0.77) | | 0.01 (−0.42, 0.43) | 0.09 (−0.41, 0.60) | | | |
| Leptin*2* | 1.13 (−0.90, 3.20) | 1.10 (−1.30, 3.55) | 0.71 (−1.09, 2.55) | 0.55 (−1.52, 2.66) | | 0.57 (−0.59, 1.74) | 0.51 (−0.87, 1.90) | | | |
| Adiponectin*2* | 0.44 (−0.24, 1.13) | 0.11 (−0.70, 0.92) | 0.37 (−0.26, 1.00) | 0.29 (−0.42, 1.00) | | 0.19 (−0.21, 0.60) | 0.13 (−0.34, 0.60) | | | |
| HOMA IR index*2* | 0.39 (−0.39, 1.17) | 0.43 (−0.49, 1.36) | −0.17 (−0.86, 0.53) | −0.11 (−0.91, 0.69) | | −0.03 (−0.48, 0.42) | 0.04 (−0.49, 0.57) | | | |
| HOMA β Index*2* | 0.79 (−0.23, 1.83) | 0.58 (−0.62, 1.80) | 0.23 (−0.52, 0.98) | 0.45 (−0.41, 1.32) | | 0.15 (−0.33, 0.64) | 0.33 (−0.24, 0.90) | | | |
| Metabolic syndrome (one criteria or more)*1* | 1.04 (1.00, 1.07) | 1.04 (0.99, 1.08) | 1.02 (0.98, 1.05) | 1.01 (0.98, 1.05) | | 1.01 (0.99, 1.03) | 1.01 (0.98, 1.03) | | | |
| *1* OR (95% CI) | | | |  |  | | | | |  |
| *2* %of change (95% CI)  Abbreviations: CI; confidence intervals, HOMA: homeostasis model assessment with HOMA IR: insulin resistance, HOMA β: pancreas beta cells activity, LDL: low density lipo-proteins; HDL: high density lipo-proteins.  Average levels of each outcome or number of cases can be found in Table 1.  Overweight or Obese: body mass index ≥ 25 kg/m^2^. Large waist: Waist circumference above 102 or 88 cm for male or female. Metabolic syndrome was defined as having at least one ATP III criteria (waist circumference > 102 cm for males and > 88 cm for females; HDL cholesterol < 1.04 mmol/L for males and < 1.30 mmol/L for females; triglycerides ≥ 1.7 mmol/L, systolic blood pressure ≥ 130 mmHg or diastolic blood pressure ≥ 85 mmHg, or fasting blood sugar ≥ 6.1 mmol/L).  Estimates are expressed by 10 pmol/g Hb increase in hemoglobin adducts of acrylamide (HbAA or HbGA or the sum of HbAA+HbGA)  Model 1 is adjusted for household income, maternal education and maternal age in pregnancy.  Model 3 is adjusted for household income, maternal education, maternal age, maternal smoking, and maternal healthy eating index in pregnancy. | | | | | | | |  |  |  |

# Table S11: Association between acrylamide and glycidamide hemoglobin adducts and standardized estimates of metabolic outcomes.

|  | n | Model 1 | Model 2 | Model 3 |
| --- | --- | --- | --- | --- |
|  |  | β (95% CI) | β (95% CI) | β (95% CI) |
| HbAA |  |  |  |  |
| Weight | 638 | −0.035 (−0.114, 0.044) | −0.048 (−0.138, 0.043) | −0.046 (−0.137, 0.046) |
| Height | 638 | −0.073 (−0.152, 0.006) | −0.049 (−0.139, 0.042) | −0.052 (−0.143, 0.040) |
| Waist circumference | 638 | 0.074 (−0.004, 0.153) | 0.046 (−0.044, 0.136) | 0.040 (−0.050, 0.131) |
| Body mass index | 638 | 0.008 (−0.069, 0.086) | −0.027 (−0.116, 0.062) | −0.023 (−0.113, 0.067) |
| Systolic blood pressure | 400 | −0.024 (−0.140, 0.091) | −0.083 (−0.219, 0.053) | −0.079 (−0.216, 0.059) |
| Diastolic blood pressure | 400 | 0.041 (−0.073, 0.156) | −0.018 (−0.152, 0.116) | −0.021 (−0.157, 0.115) |
| Triglycerides | 400 | −0.001 (−0.129, 0.126) | −0.041 (−0.191, 0.109) | −0.037 (−0.189, 0.115) |
| Total cholesterol | 400 | 0.076 (−0.034, 0.186) | 0.075 (−0.054, 0.205) | 0.060 (−0.071, 0.191) |
| LDL cholesterol | 400 | 0.121 (0.006, 0.236) | 0.135 (0.000, 0.270) | 0.118 (−0.019, 0.254) |
| HDL cholesterol | 400 | −0.054 (−0.178, 0.071) | −0.063 (−0.209, 0.083) | −0.072 (−0.220, 0.077) |
| Blood sugar | 400 | 0.015 (−0.095, 0.125) | 0.038 (−0.091, 0.167) | 0.057 (−0.074, 0.187) |
|  |  |  |  |  |
| HbGA |  |  |  |  |
| Weight | 241 | −0.065 (−0.198, 0.067) | −0.067 (−0.218, 0.083) | −0.065 (−0.216, 0.086) |
| Height | 241 | −0.082 (−0.208, 0.045) | −0.041 (−0.186, 0.103) | −0.046 (−0.190, 0.099) |
| Waist circumference | 241 | 0.046 (−0.101, 0.193) | 0.010 (−0.155, 0.175) | 0.009 (−0.157, 0.175) |
| Body mass index | 241 | −0.032 (−0.168, 0.105) | −0.068 (−0.222, 0.086) | −0.061 (−0.215, 0.093) |
| Systolic blood pressure | 148 | −0.141 (−0.306, 0.025) | −0.175 (−0.364, 0.015) | −0.177 (−0.369, 0.015) |
| Diastolic blood pressure | 148 | −0.038 (−0.219, 0.144) | −0.090 (−0.299, 0.119) | −0.101 (−0.312, 0.110) |
| Triglycerides | 148 | 0.025 (−0.158, 0.209) | −0.018 (−0.231, 0.195) | 0.000 (−0.215, 0.214) |
| Total cholesterol | 148 | 0.198 (0.042, 0.354) | 0.192 (0.014, 0.371) | 0.179 (−0.001, 0.358) |
| LDL cholesterol | 148 | 0.249 (0.090, 0.408) | 0.263 (0.080, 0.445) | 0.247 (0.063, 0.430) |
| HDL cholesterol | 148 | −0.001 (−0.184, 0.181) | −0.024 (−0.231, 0.182) | −0.039 (−0.247, 0.169) |
| Blood sugar | 148 | −0.104 (−0.248, 0.039) | −0.110 (−0.266, 0.046) | −0.118 (−0.275, 0.040) |

Abbreviations: HOMA: homeostasis model assessment with HOMA IR: insulin resistance, HOMA β: pancreas beta cells activity, LDL: low density lipo-proteins; HDL: high density lipo-proteins. HbAA: Hemoglobin adducts of acrylamide; HbGA: Hemoglobin adducts glycidamide.

Model 1: adjusted for household income, maternal education and maternal age in pregnancy.

Model 2: Model 1 further adjusted for maternal smoking in pregnancy.

Model 3 (final model): Model 2 further adjusted for maternal healthy eating index in pregnancy.

Standardized estimates are calculated using the following equation: $\beta_{std}\text{=}\frac{\beta\text{*}SD(exposure)}{SD(outcome)}$.

They are only calculated for linear models without natural log transformation

# Table S12: Summary of interactions between prenatal exposure to acrylamide and metabolic health at 20 years of age and mother education (high vs low), mother smoking (yes vs no) and offspring sex (female vs male).

|  |  | **Mother education** |  | **Mother smoking** |  | **Offspring sex** |  |
| --- | --- | --- | --- | --- | --- | --- | --- |
| Weight | β_1_ | −0.03 (−0.2, 0.2) | 0.486 | −0.05 (−0.3, 0.2) | 0.759 | −0.001 (−0.2, 0.2) | <0.001 |
|  | β_2_ | −4 (−40, 30) |  | 10 (−30, 60) |  | −100 (−100, −90) |  |
|  | β_1_ x β_2_ | −0.1 (−0.4, 0.2) |  | −0.05 (−0.4, 0.3) |  | −0.1 (−0.4, 0.1) |  |
| Height | β_1_ | −0.05 (−0.2, 0.1) | 0.634 | −0.04 (−0.3, 0.2) | 0.707 | −0.07 (−0.2, 0.03) | <0.001 |
|  | β_2_ | 4 (−30, 30) |  | −5 (−40, 30) |  | −100 (−200, −100) |  |
|  | β_1_ x β_2_ | −0.05 (−0.3, 0.2) |  | −0.05 (−0.3, 0.2) |  | 0.06 (−0.08, 0.2) |  |
| Waist circumference | β_1_ | 0.2 (−0.01, 0.3) | 0.085 | 0.05 (−0.2, 0.3) | 0.934 | 0.1 (−0.05, 0.3) | <0.001 |
|  | β_2_ | 6 (−20, 40) |  | 10 (−20, 40) |  | −40 (−70, −10) |  |
|  | β_1_ x β_2_ | −0.2 (−0.4, 0.03) |  | 0.01 (−0.3, 0.3) |  | −0.09 (−0.3, 0.1) |  |
| Waist circumference above 102 or 88^1^ | β_1_ | 1.01 (9.64 × 10^−1^, 1.06) | 0.385 | 9.80 × 10^−1^ (8.80 × 10^−1^, 1.06) | 0.619 | 1.04 (9.73 × 10^−1^, 1.11) | <0.001 |
|  | β_2_ | 2.58 × 10^−1^ (6.38 × 10^−6^, 1.60 × 10^4^) |  | 7.45 × 10^1^ (3.67 × 10^−4^, 9.25 × 10^6^) |  | 7.41 × 10^10^ (3.16 × 10^5^, 3.89 × 10^16^) |  |
|  | β_1_ x β_2_ | 9.65 × 10^−1^ (8.83 × 10^−1^, 1.04) |  | 1.03 (9.33 × 10^−1^, 1.15) |  | 9.34 × 10^−1^ (8.67 × 10^−1^, 1.01) |  |
| Body mass index | β_1_ | 0.002 (−0.05, 0.06) | 0.596 | −0.006 (−0.08, 0.06) | 0.895 | 0.02 (−0.03, 0.07) | 0.007 |
|  | β_2_ | −2 (−10, 7) |  | 5 (−5, 20) |  | −0.4 (−9, 8) |  |
|  | β_1_ x β_2_ | −0.02 (−0.09, 0.05) |  | −0.006 (−0.09, 0.08) |  | −0.06 (−0.1, 0.01) |  |
| Overweight or Obese^1^ | β_1_ | 9.88 × 10^−1^ (9.45 × 10^−1^, 1.03) | 0.805 | 9.72 × 10^−1^ (9.01 × 10^−1^, 1.04) | 0.68 | 1.00 (9.54 × 10^−1^, 1.04) | 0.48 |
|  | β_2_ | 4.32 × 10^−2^ (1.37 × 10^−5^, 1.62 × 10^2^) |  | 1.86 × 10^1^ (1.48 × 10^−3^, 2.00 x 10^5^) |  | 1.83 × 10^1^ (6.91 × 10^−3^, 4.92 × 10^4^) |  |
|  | β_1_ x β_2_ | 9.92 × 10^−1^ (9.28 × 10^−1^, 1.06) |  | 1.02 (9.42 × 10^−1^, 1.11) |  | 9.66 × 10^−1^ (9.08 × 10^−1^, 1.03) |  |
| Systolic blood pressure | β_1_ | −0.1 (−0.4, 0.2) | 0.881 | −0.1 (−0.5, 0.2) | 0.982 | −0.007 (−0.3, 0.3) | <0.001 |
|  | β_2_ | 8 (−40, 50) |  | 20 (−30, 70) |  | −100 (−200, −90) |  |
|  | β_1_ x β_2_ | −0.03 (−0.4, 0.3) |  | 0.005 (−0.4, 0.5) |  | −0.02 (−0.3, 0.3) |  |
| Diastolic blood pressure | β_1_ | −0.07 (−0.2, 0.09) | 0.183 | 0.008 (−0.2, 0.2) | 0.763 | −0.09 (−0.3, 0.1) | 0.076 |
|  | β_2_ | −20 (−50, 5) |  | 20 (−20, 50) |  | 5 (−20, 30) |  |
|  | β_1_ x β_2_ | 0.2 (−0.07, 0.4) |  | −0.04 (−0.3, 0.2) |  | 0.09 (−0.1, 0.3) |  |
| Triglycerides | β_1_ | −0.006 (−0.02, 0.005) | 0.331 | −0.01 (−0.03, 0.002) | 0.091 | −0.002 (−0.02, 0.01) | 0.002 |
|  | β_2_ | −0.8 (−3, 1) |  | −1 (−3, 1) |  | 2 (0.02, 4) |  |
|  | β_1_ x β_2_ | 0.008 (−0.008, 0.02) |  | 0.02 (−0.003, 0.03) |  | −0.002 (−0.02, 0.01) |  |
| Total cholesterol | β_1_ | −0.003 (−0.02, 0.02) | 0.077 | −0.006 (−0.04, 0.02) | 0.279 | 0.02 (−0.008, 0.04) | <0.001 |
|  | β_2_ | −2 (−6, 1) |  | −2 (−7, 2) |  | 7 (4, 10) |  |
|  | β_1_ x β_2_ | 0.03 (−0.003, 0.06) |  | 0.02 (−0.02, 0.05) |  | −0.02 (−0.05, 0.008) |  |
| LDL cholesterol | β_1_ | 0.004 (−0.01, 0.02) | 0.096 | 0.003 (−0.02, 0.03) | 0.368 | 0.02 (0.003, 0.05) | 0.013 |
|  | β_2_ | −2 (−5, 1) |  | −2 (−5, 2) |  | 4 (0.9, 7) |  |
|  | β_1_ x β_2_ | 0.02 (−0.004, 0.04) |  | 0.01 (−0.02, 0.04) |  | −0.02 (−0.04, 0.004) |  |
| HDL cholesterol | β_1_ | −0.004 (−0.01, 0.004) | 0.62 | −0.003 (−0.01, 0.009) | 0.913 | −0.005 (−0.01, 0.004) | <0.001 |
|  | β_2_ | 0.03 (−1, 1) |  | 0.07 (−2, 2) |  | 3 (1, 4) |  |
|  | β_1_ x β_2_ | 0.003 (−0.008, 0.01) |  | −0.0007 (−0.01, 0.01) |  | −0.0005 (−0.01, 0.01) |  |
| Blood sugar | β_1_ | 0.003 (−0.009, 0.01) | 0.592 | 0.01 (−0.003, 0.03) | 0.196 | 0.008 (−0.006, 0.02) | <0.001 |
|  | β_2_ | −0.03 (−2, 2) |  | 1 (−1, 3) |  | −3 (−5, −1) |  |
|  | β_1_ x β_2_ | 0.004 (−0.01, 0.02) |  | −0.01 (−0.03, 0.007) |  | −0.001 (−0.02, 0.01) |  |
| Insulin^2^ | β_1_ | 8.08 × 10^−2^ (−9.85 × 10^−1^, 1.16) | 0.375 | 1.05 (−4.63 × 10^−1^, 2.58) | 0.279 | 5.34 × 10^−1^ (−8.19 × 10^−1^, 1.91) | 0.321 |
|  | β_2_ | −9.11 × 10^1^ (−9.86 × 10^1^, −4.48 × 10^1^) |  | 1.49 × 10^2^ (−7.02 × 10^1^, 1.98 × 10^3^) |  | 1.67 × 10^2^ (−5.49 × 10^1^, 1.49 × 10^3^) |  |
|  | β_1_ x β_2_ | 6.71 × 10^−1^ (−8.09 × 10^−1^, 2.17) |  | −9.84 × 10^−1^ (−2.74, 8.07 × 10^−1^) |  | −3.20 × 10^−1^ (−1.81, 1.19) |  |
| Leptin^2^ | β_1_ | 1.56 × 10^−1^ (−2.78, 3.18) | 0.233 | 1.66 (−2.48, 5.98) | 0.746 | 7.49 × 10^−1^ (−1.90, 3.47) | <0.001 |
|  | β_2_ | −9.90 × 10^1^ (−1.00 × 10^2^, 6.07 × 10^1^) |  | 8.75 × 10^1^ (−9.95 × 10^1^, 6.62 × 10^4^) |  | 1.39 × 10^10^ (4.17 × 10^8^, 4.63 × 10^11^) |  |
|  | β_1_ x β_2_ | 2.53 (−1.60, 6.83) |  | −8.13 × 10^−1^ (−5.61, 4.22) |  | −1.44 (−4.33, 1.53) |  |
| Adiponectin^2^ | β_1_ | −6.76 × 10^−1^ (−1.65, 3.07 × 10^−1^) | 0.009 | 1.01 (−3.92 × 10^−1^, 2.43) | 0.123 | −4.92 × 10^−1^ (−1.63, 6.63 × 10^−1^) | <0.001 |
|  | β_2_ | −8.81 × 10^1^ (−9.78 × 10^1^, −3.58 × 10^1^) |  | 5.88 × 10^2^ (−3.80, 4.82 × 10^3^) |  | 1.55 × 10^3^ (2.62 × 10^2^, 7.41 × 10^3^) |  |
|  | β_1_ x β_2_ | 1.83 (4.56 × 10^−1^, 3.23) |  | −1.30 (−2.92, 3.54 × 10^−1^) |  | 4.98 × 10^−1^ (−7.85 × 10^−1^, 1.80) |  |
| HOMA IR index^2^ | β_1_ | 5.59 × 10^−3^ (−1.32 × 10^−2^, 2.43 × 10^−2^) | <0.001 | 3.75 × 10^−2^ (1.12 × 10^−2^, 6.38 × 10^−2^) | 0.008 | 1.30 × 10^−2^ (−1.08 × 10^−2^, 3.68 × 10^−2^) | 0.008 |
|  | β_2_ | −4.44 (−7.64, −1.24) |  | 2.47 (−1.24, 6.17) |  | 2.73 × 10^−1^ (−2.86, 3.40) |  |
|  | β_1_ x β_2_ | 1.83 × 10^−2^ (−7.60 × 10^−3^, 4.42 × 10^−2^) |  | −3.59 × 10^−2^ (−6.72 × 10^−2^, −4.62 × 10^−3^) |  | −4.34 × 10^−4^ (−2.70 × 10^−2^, 2.61 × 10^−2^) |  |
| HOMA β Index^2^ | β_1_ | 7.32 × 10^−2^ (−1.46, 1.60) | 0.032 | 9.20 × 10^−1^ (−1.23, 3.07) | 0.125 | −5.28 × 10^−2^ (−1.93, 1.83) | 0.125 |
|  | β_2_ | −1.99 × 10^2^ (−4.60 × 10^2^, 6.22 × 10^1^) |  | 1.31 × 10^2^ (−1.72 × 10^2^, 4.34 × 10^2^) |  | 3.12 × 10^2^ (6.53 × 10^1^, 5.59 × 10^2^) |  |
|  | β_1_ x β_2_ | 5.33 × 10^−2^ (−2.06, 2.17) |  | −1.10 (−3.66, 1.46) |  | −3.41 × 10^−2^ (−2.13, 2.06) |  |
| Metabolic syndrome (one criteria or more)^1^ | β_1_ | 1.01 (9.59 × 10^−1^, 1.06) | 0.069 | 1.07 (9.94 × 10^−1^, 1.15) | 0.292 | 1.00 (9.33 × 10^−1^, 1.07) | 0.047 |
|  | β_2_ | 8.69 × 10^−5^ (1.11 × 10^−8^, 6.48 × 10^−1^) |  | 2.13 × 10^2^ (6.58 × 10^−3^, 6.38 × 10^6^) |  | 1.08 (1.36 × 10^−4^, 8.71 × 10^3^) |  |
|  | β_1_ x β_2_ | 1.07 (9.95 × 10^−1^, 1.15) |  | 9.55 × 10^−1^ (8.76 × 10^−1^, 1.04) |  | 1.05 (9.72 × 10^−1^, 1.13) |  |

Abbreviations: HOMA: homeostasis model assessment with HOMA IR: insulin resistance, HOMA β: pancreas beta cells activity, LDL: low density lipo-proteins; HDL: high density lipo-proteins. β1: HbAA levels. β2: coefficient for mother education (high, with low as reference), mother smoking (yes, with no as reference) or offspring sex (female with male). β1 x β2: interaction coefficient.

^1^ OR (95% CI). ^2^ Natural logarithm transformed outcome; estimates expressed as percentage of change (95% CI).

Linear estimates are expressed by 10 pmol/g increase in hemoglobin adducts of acrylamide.

Estimates are from models adjusted for household income, maternal education, maternal age, maternal smoking, and maternal healthy eating index in pregnancy.

# Figures

## Figure S*1*. Flowchart of the study populations.


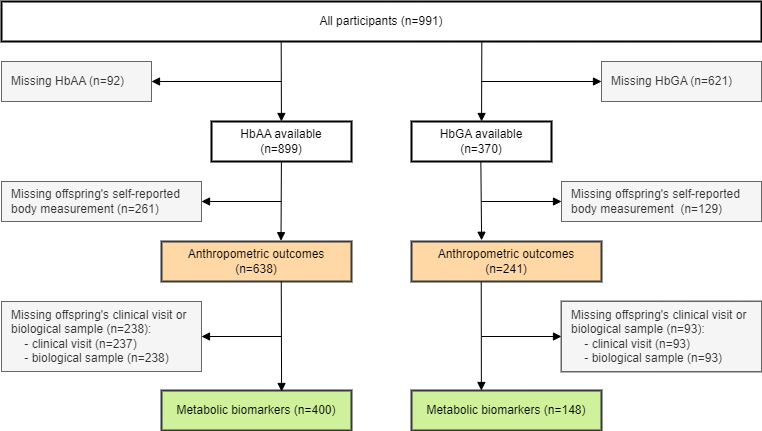


## Figure S*2*. Directed acyclic graph of the association between prenatal exposure to acrylamide and metabolic status at age 20.


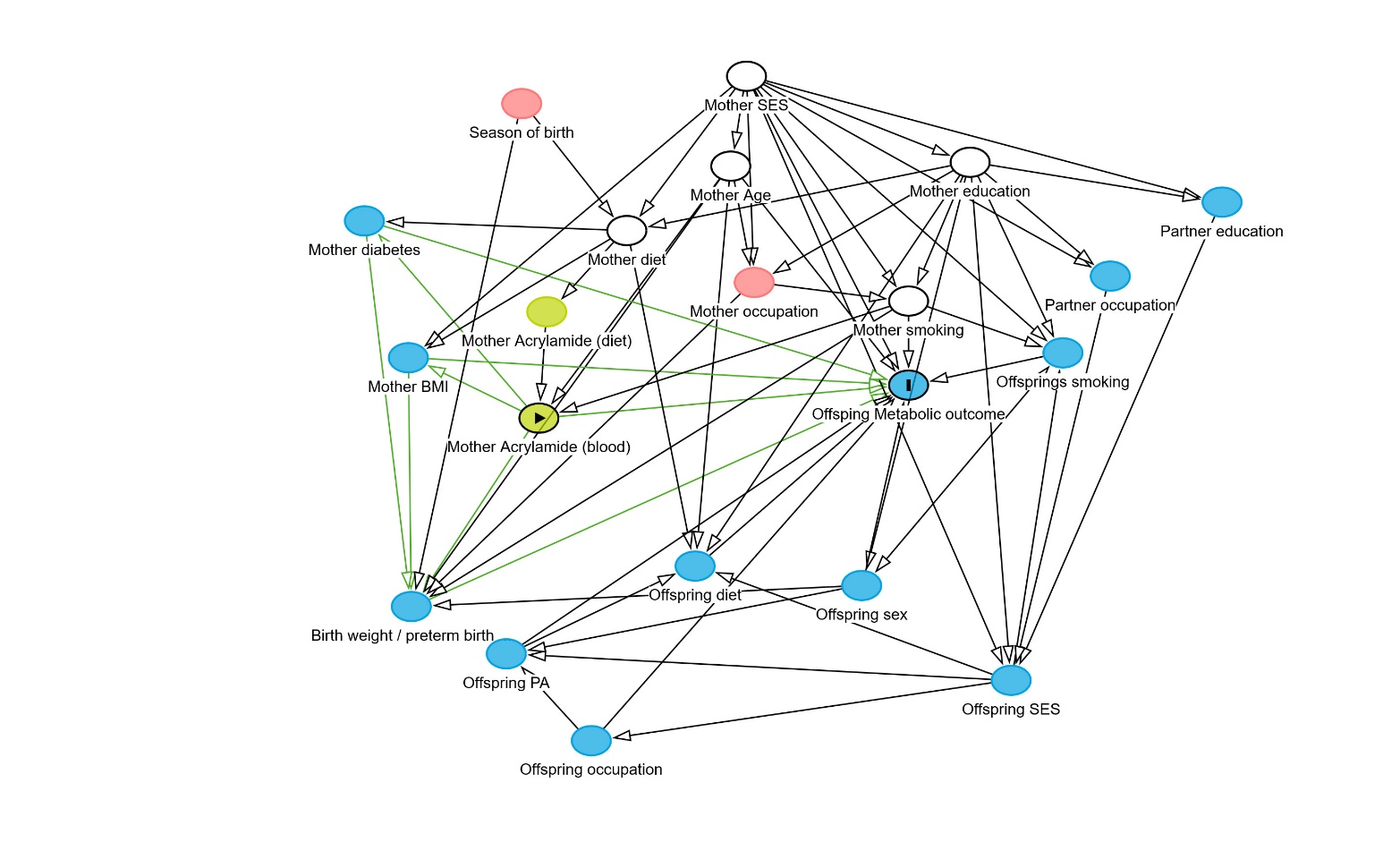


Green arrow: Exposure, Blue I: Outcome, Blue circles: Ancestors of outcome, Green circles: Ancestors of exposure, White circles: Variables adjusted in our model, Red: Unadjusted confounders. All paths are black indicating no residual confounding.

## Figure S*3*. Distribution of hemoglobin adducts of acrylamide and glycidamide for all and by maternal smoking status.


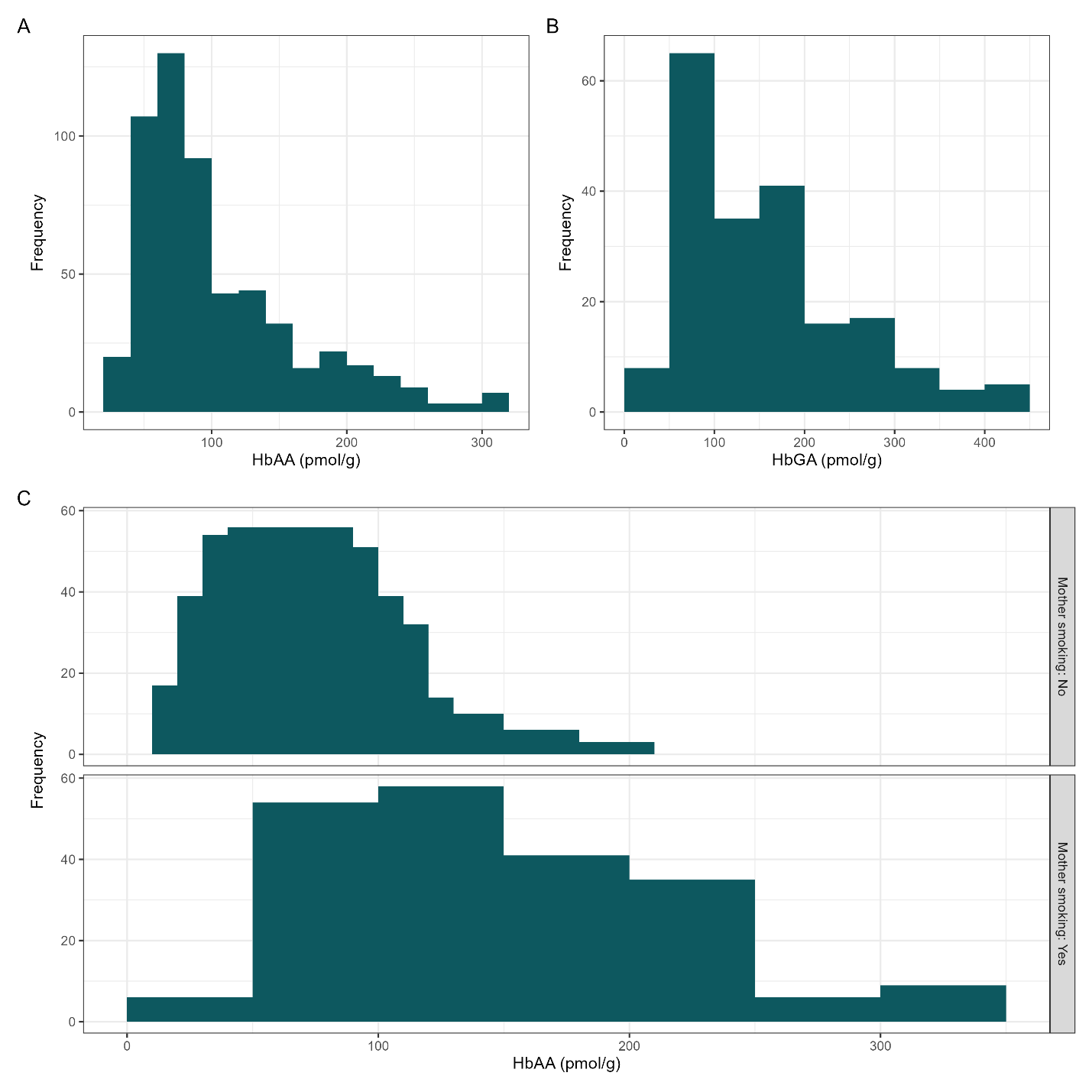


A: Distribution of HbAA.

B: Distribution of HbGA.

C: Distribution of HbAA among non-smoking and smoking mothers during pregnancy.

HbAA: Hemoglobin adducts of acrylamide; HbGA: Hemoglobin adducts of glycidamide

## Figure S*4*. Correlation matrix between HbAA, HbGA and metabolic health outcomes


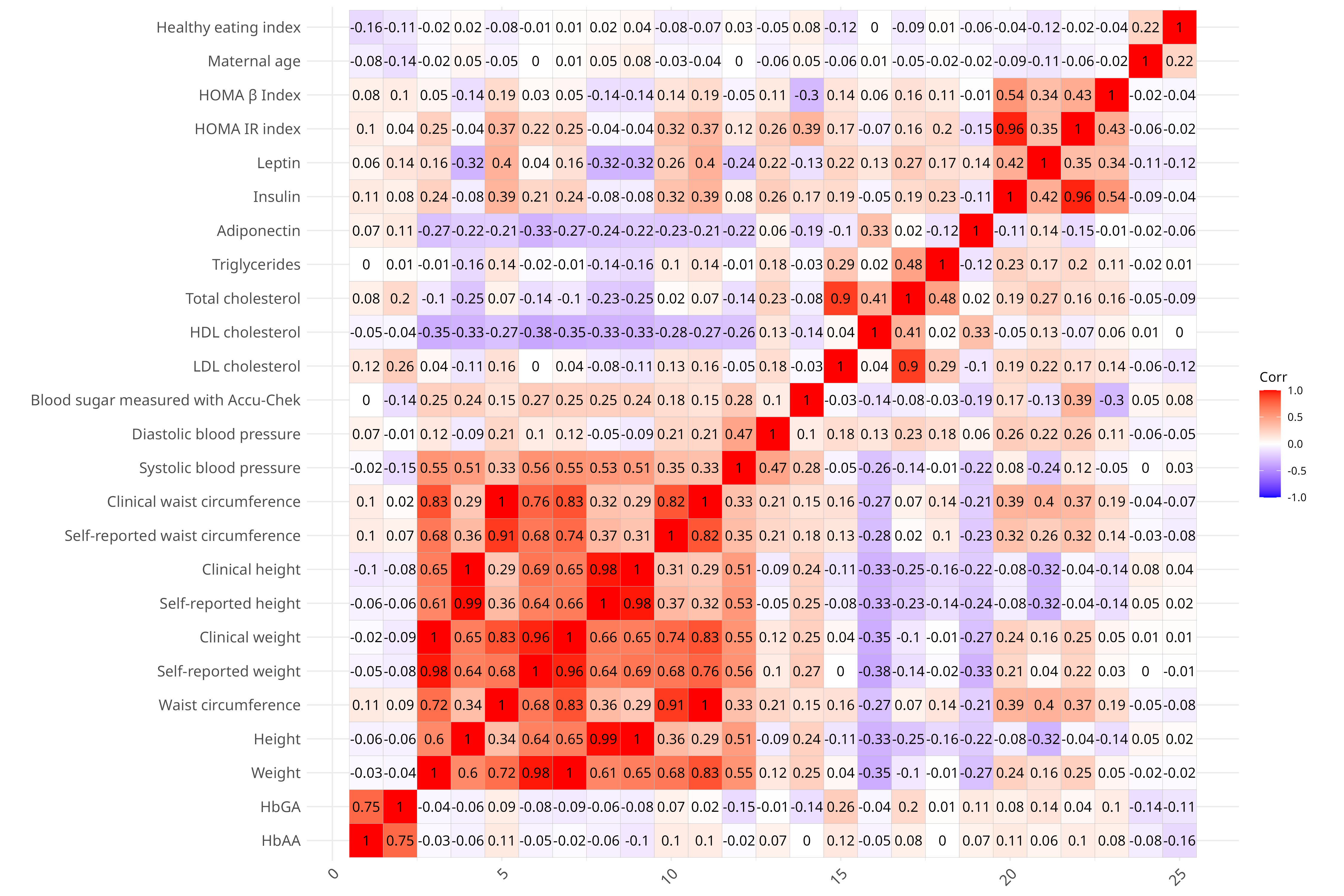


## Figure S*5*. Dose-response curves of the associations between prenatal exposure to acrylamide and additional metabolic outcomes at 20 years of age.


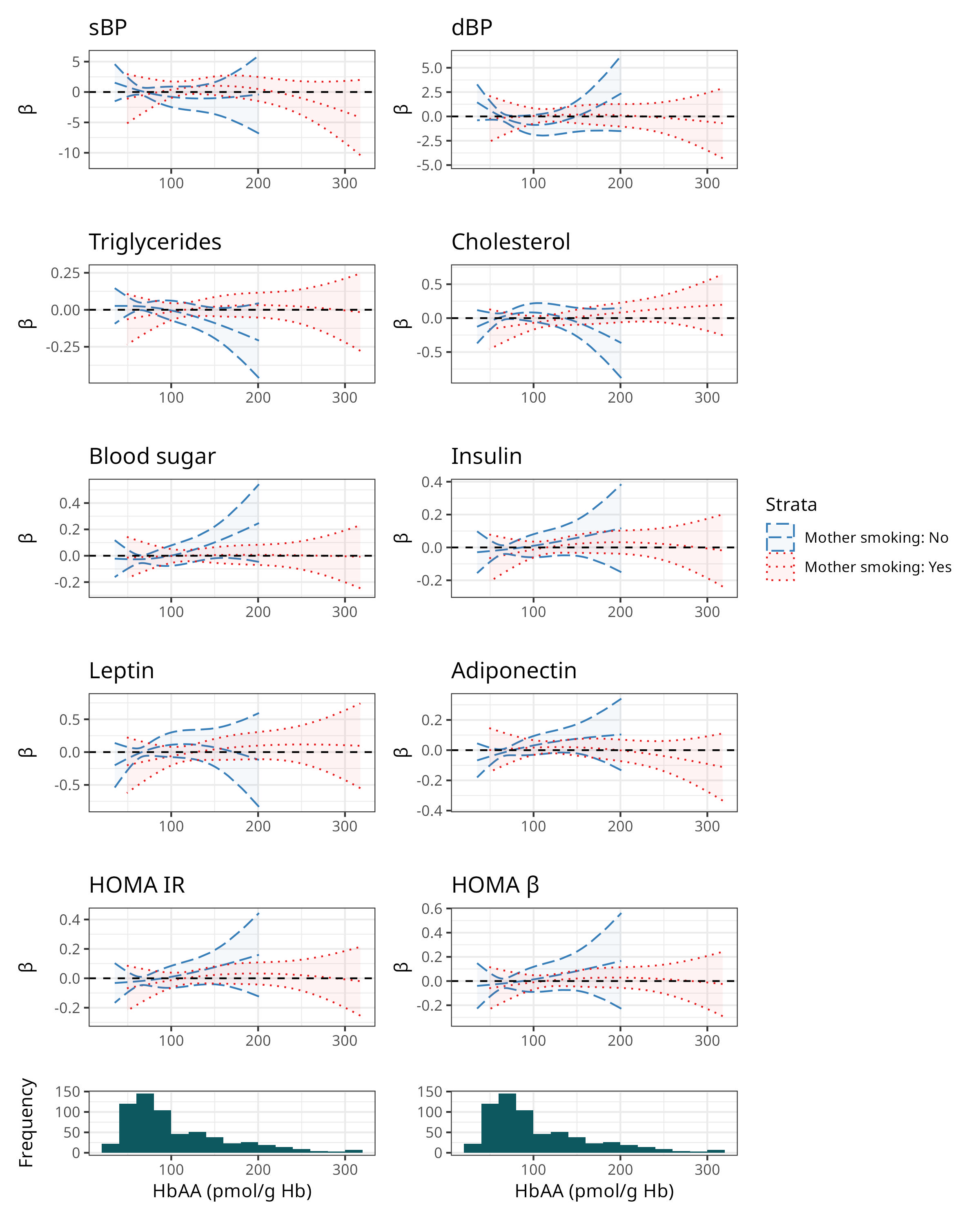


The curves are expressed relative to minimum exposure with 2df (lowest AIC) from models adjusted for household income, maternal education, maternal age, maternal smoking, and maternal healthy eating index in pregnancy.

The models stratified by smoking status are restricted to non-smoking mother or smoking mother offspring pairs and adjusted for household income, maternal education, maternal age, and maternal healthy eating index in pregnancy.

The solid lines show the association for all participants, the dashed lines for non-smokers mothers and the dotted lines for smokers.

sBP: Systolic blood pressure (mmHg); dBP: Diastolic blood pressure (mmHg); HOMA: homeostasis model assessment with HOMA IR: insulin resistance, HOMA β: pancreas beta cells activity; HbAA: Hemoglobin adducts of acrylamide; HbGA: Hemoglobin adducts of glycidamide

## Figure S*6*. Dose-response curves of the associations between prenatal exposure to glycidamide and metabolic status at 20 years of age.


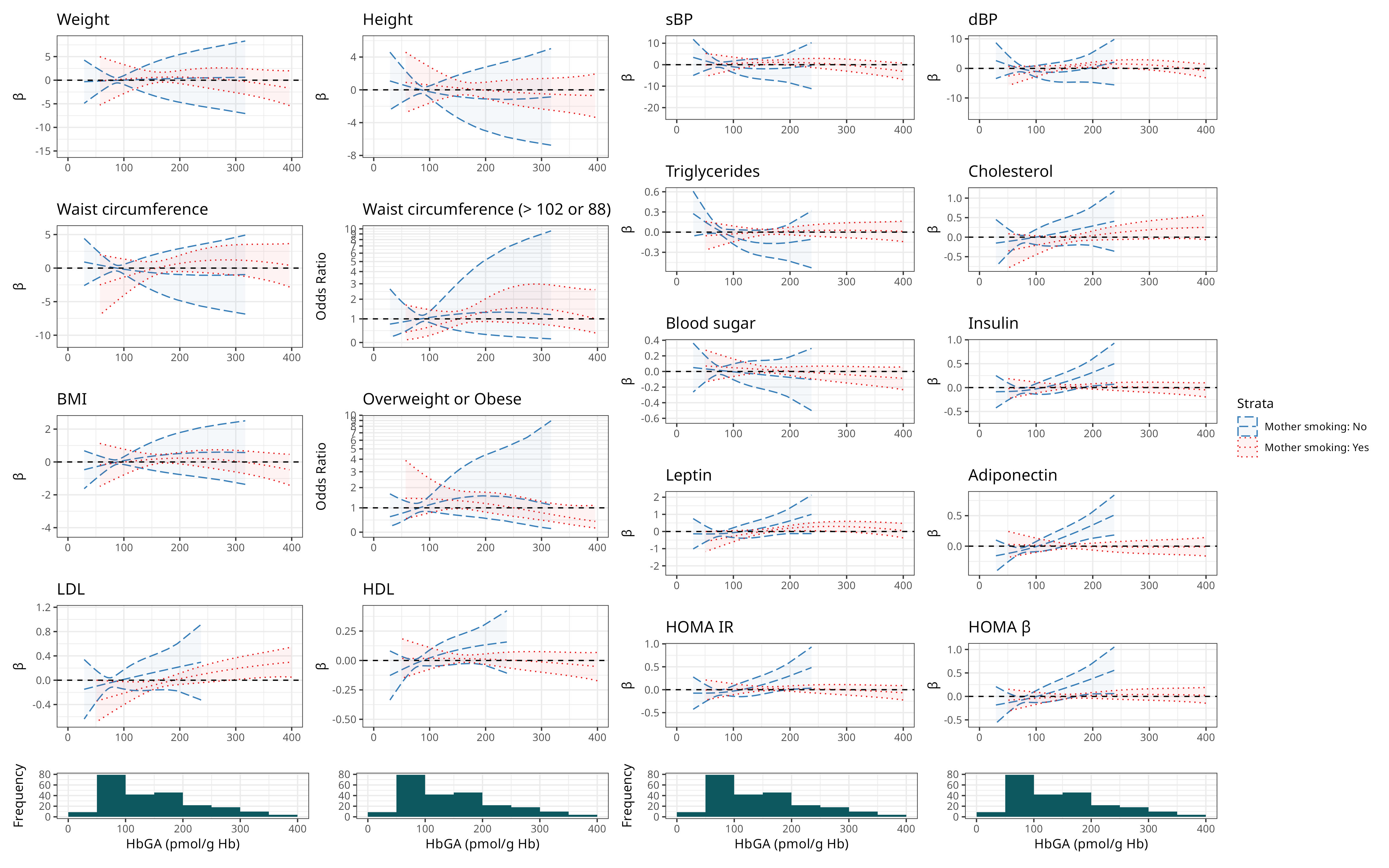
The curves are expressed relative to minimum exposure with 2df (lowest AIC) from models adjusted for household income, maternal education, maternal age, maternal smoking, and maternal healthy eating index in pregnancy. The models stratified by smoking status are restricted to non-smoking mother or smoking mother offspring pairs and adjusted for household income, maternal education, maternal age, and maternal healthy eating index in pregnancy. The solid lines show the association for all participants, the dashed lines for non-smokers mothers and the dotted lines for smokers.

BMI: Body mass index; LDL: low density lipoprotein; HDL: high density lipoprotein; sBP: Systolic blood pressure (mmHg); dBP: Diastolic blood pressure (mmHg); HOMA: homeostasis model assessment with HOMA IR: insulin resistance, HOMA β: pancreas beta cells activity. HbAA: Hemoglobin adducts of acrylamide; HbGA: Hemoglobin adducts of glycidamide
